# A Functionally Conserved Enhancer is Critical for *PGC1A* Expression and Thermogenesis in Brown Fat

**DOI:** 10.1002/advs.202518153

**Published:** 2026-03-23

**Authors:** Duo Su, Haosen Yang, Xiaokai Li, Tingting Jiang, Siyuan Zhan, Tao Zhong, Jiazhong Guo, Yan Wang, Li Li, Hongping Zhang, Linjie Wang

**Affiliations:** ^1^ Key Laboratory of Livestock and Poultry Multi‐Omics Ministry of Agriculture and Rural Affairs College of Animal Science and Technology Sichuan Agricultural University Chengdu Sichuan P. R. China; ^2^ Farm Animal Germplasm Resources and Biotech Breeding Key Laboratory of Sichuan Province College of Animal Science and Technology Sichuan Agricultural University Chengdu Sichuan P. R. China; ^3^ Key Laboratory of Agricultural Bioinformatics Ministry of Education Sichuan Agricultural University Chengdu Sichuan P. R. China; ^4^ Chongqing Academy of Animal Sciences Chongqing P. R. China; ^5^ National Center of Technology Innovation for Pigs Chongqing P. R. China

**Keywords:** brown adipose tissue, conserved enhancer, epigenomics, PGC1A transcription regulation, thermogenesis

## Abstract

Brown adipose tissue (BAT) undergoes progressive age‐related involution into white adipose tissue (WAT), yet the epigenetic mechanisms underlying BAT whitening remain unclear. This study investigates the transcriptomic dynamics of goat perirenal adipose tissue (PRAT) across four postnatal stages and profiles active histone modifications and chromatin accessibility in PRAT at postnatal day 1 (BAT) and day 90 (WAT). PRAT whitening coincides with reduced enrichment of active histone marks and decreased chromatin accessibility at genes involved in thermogenesis and mitochondrial function, critical for maintaining brown adipocyte identity. Integration of high‐resolution *PGC1A* chromatin interaction mapping identifies two BAT‐specific active enhancers, PGC1A‐En1 and PGC1A‐En2, which exhibit additive effects and are further activated under cold stimulation. Functional repression of PGC1A‐En1 and/or PGC1A‐En2 in goat brown adipocytes reduces *PGC1A* expression and impairs thermogenic capacity, highlighting the pivotal role of PGC1A‐En1. Consistently, lentivirus‐mediated repression of the conserved mouse Pgc1a‐En1 in vivo decreases *Pgc1a* expression and attenuates cold‐induced thermogenesis in BAT. Notably, the conserved PGC1A‐hEn1 also functions as a critical regulatory element required for *PGC1A* expression and thermogenic activation in human brown adipocytes. These findings elucidate epigenetic mechanisms controlling BAT activity and identify conserved enhancer elements as potential therapeutic targets for metabolic disorders.

## Graphical abstract

An evolutionarily conserved enhancer controls *PGC1A* expression and thermogenic activation in brown adipose tissue. Integrative multi‐omics analyses identify two BAT‐specific enhancers, with PGC1A‐En1 functioning as the dominant regulatory element that maintains thermogenic capacity across species. Cooperative binding of ERRA and CBP activates this enhancer through a conserved motif to promote brown fat thermogenesis.

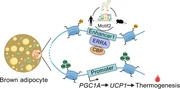

## 1 Introduction

Adipose tissue is a highly plastic organ that plays an essential role in regulating whole‐body metabolism and energy homeostasis [1]. In mammals, adipose tissue is generally classified into three types: white adipose tissue (WAT), brown adipose tissue (BAT), and beige adipose tissue. WAT primarily functions as an energy reservoir, storing excess energy in the form of triglycerides, whereas BAT is specialized for non‐shivering thermogenesis, dissipating energy as heat to maintain body temperature [2, 3]. In humans, the majority of BAT develops prenatally and is mature and fully functional at birth, but most of it is gradually replaced by WAT with increasing age [2]. This age‐related transformation, known as BAT whitening, is characterized by a white adipocyte‐like appearance with attenuated UCP1 expression in BAT [4]. Accumulated evidence has shown that functionally active BAT is negatively correlated with body mass index [5, 6]. Moreover, in vivo studies suggest that stimulation of BAT recruitment and activation can correct dyslipidemia and protect against obesity‐related metabolic disorders [7, 8, 9, 10]. However, BAT is detected in only approximately 10% of individuals and decreases with age [11, 12]. Therefore, feasible strategies to promote BAT development or expand BAT depots may aid in the treatment of metabolic diseases.

Unlike humans, mice maintained at standard room temperature exhibited limited progressive involution of BAT with age. While both rodents and humans possess BAT, these depots persist throughout life in mice, whereas they decline with age in humans [13, 14]. Therefore, mice may not serve as the most suitable model for elucidating the mechanisms underlying the involution of human BAT. In large ruminants, such as goats, BAT depots are fully mature and functional at birth, playing a crucial role in thermoregulation [15]. These depots gradually transition into WAT during the postnatal period, at a developmental stage comparable to when BAT largely disappears in humans [11, 16]. Previous studies have shown that thermogenic brown‐like adipocytes in goat perirenal adipose tissue (PRAT) progressively transform into white‐like adipocytes within weeks after birth [17, 18]. Despite extensive research on goat PRAT whitening at the morphological and transcriptomic levels, the underlying epigenetic mechanisms regulating this transition remain poorly understood.

Enhancers are key *cis*‐regulatory elements that enhance the transcription of their target genes by physically interacting with distant promoters [19]. The precise spatial and temporal regulation of gene expression during development depends on the specific binding of transcription factors (TFs) to enhancers, which is affected by chromatin state and accessibility [20, 21]. Circular chromosome conformation capture sequencing (4C‐seq), a three‐dimensional genome mapping technique that interrogates long‐range chromatin interactions genome‐wide from a predefined genomic locus as the viewpoint [22, 23, 24]. Active enhancers are characterized by regions of open chromatin flanked by nucleosomes enriched in histone 3 lysine 27 acetylation (H3K27ac), whereas actively transcribed genes are marked by histone 3 lysine 4 trimethylation (H3K4me3) [25, 26]. PGC1A is a crucial transcriptional coactivator that activates several key components of the adaptive thermogenic gene program in brown fat [27, 28]. Mice lacking PGC1A exhibited cold sensitivity, reduced UCP1 expression, and abnormal BAT morphology characterized by the accumulation of large lipid droplets resembling WAT [29]. Although the loss of PGC1A does not impair differentiation of brown adipocytes, it significantly reduces the induction of key thermogenic genes, such as *Ucp1* and *Dio2* [30]. Elucidating the molecular mechanisms governing *PGC1A* expression will provide important insights into promoting BAT thermogenesis for the prevention of obesity and related metabolic disorders.

In this study, we investigated the transcriptomic dynamics of goat PRAT across four postnatal developmental stages and profiled active histone modifications, chromatin accessibility, and *PGC1A* chromatin interactions during PRAT whitening. We identified a functionally conserved enhancer essential for *PGC1A* expression and thermogenesis in brown fat. Elucidating the regulatory mechanisms underlying BAT activation may reveal novel therapeutic targets for metabolic dysfunction.

## 2 Results

### 2.1 Dynamic Transcriptome Changes During PRAT Whitening in Goats

**FIGURE 1:**
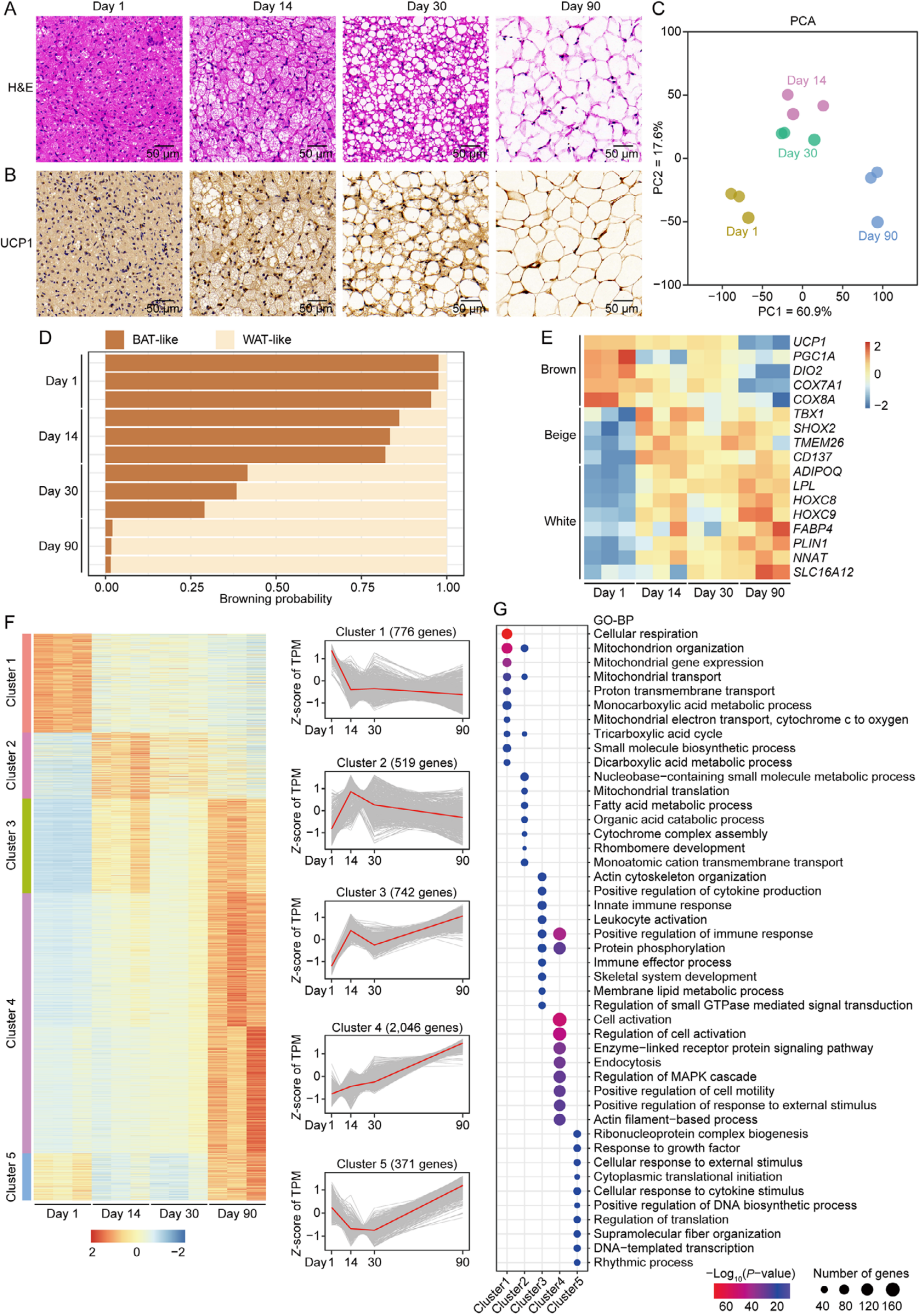
Characterization of the postnatal transformation from brown to white adipose tissue. (A,B) Hematoxylin and eosin (H&E) staining (A) and uncoupling protein 1 (UCP1) immunohistochemistry (B) of perirenal adipose tissue (PRAT) at postnatal days 1, 14, 30, and 90. Scale bar = 50 µm. (C) Principal component analysis (PCA) plot of normalized RNA‐seq data from PRAT at postnatal days 1, 14, 30, and 90 (*n* = 3). D) Browning probability of PRAT estimated using the Profiling of Fat Tissue Types (ProFAT) database at postnatal days 1, 14, 30, and 90 (*n* = 3). (E) Heatmap showing the expression levels (z‐scores of TPM) of adipocyte marker genes at postnatal days 1, 14, 30, and 90 (*n* = 3). (F) Gene expression clusters identified by *k*‐means clustering. Left: Heatmap based on z‐scores of TPM values. Right: Temporal expression trends of the five clusters. Red lines indicate average expression levels; grey lines show individual gene expression trajectories during PRAT whitening. (G) Top ten enriched Gene Ontology Biological Process (GO‐BP) terms for genes in each cluster.

To characterize PRAT whitening in goats, we collected PRAT samples on postnatal days 1, 14, 30, and 90, followed by histological analysis and UCP1 immunohistochemical (IHC) staining. H&E staining revealed typical BAT morphology in PRAT on day 1 (Figure 1A). By days 14 to 30, PRAT appeared as a mixture of brown adipocytes with multilocular lipid droplets and white adipocytes with large unilocular lipid droplets (Figure 1A). By day 30, white adipocyte morphology predominated, with complete conversion to WAT‐like characteristics observed by day 90 (Figure 1A). We then examined UCP1 expression by IHC. At 1 day, UCP1 was more abundant in PRAT, gradually decreasing by days 14 to 30, and was nearly absent by day 90 (Figure 1B).

To verify the morphological observations, we generated a total of 214.49 Gb of high‐quality bulk RNA‐seq data, with three biological replicates at different ages (∼17.87 Gb per sample) (Table S1). The high Pearson correlation coefficient (*r* > 0.9) between biological replicates indicated a high reproducibility (Figure S1A). Principal component analysis (PCA) revealed a distinct separation between PRAT samples from day 1 and day 90, while there was no obvious difference between samples from day 14 and day 30 (Figure 1C). Additionally, we performed differential gene expression analysis across different ages (Table S2 and Figure S1B) and found that the comparison between day 1 and day 90 had the greatest number of differentially expressed genes (DEGs), whereas substantially fewer DEGs were identified between day 14 and day 30 (Figure S1C). Functional enrichment analysis by Metascape revealed that early DEGs were enriched in processes such as response to hormone, mitochondrion organization, mitochondrial transport, and the tricarboxylic acid cycle (Figure S1D), all of which are related to BAT function. To assess the browning probability of PRAT at different ages, we used the computational tool ProFat [31], which showed that by day 90, PRAT had largely lost its browning capacity, exhibiting increased characteristics of WAT (Figure 1D). This result was consistent with types of adipose tissue‐specific gene expression patterns. Brown adipocyte markers, including *UCP1*, *PGC1A*, *DIO2*, *COX7A1*, and *COX8A* were dramatically downregulated from day 1 to day 90 in PRAT (Figure 1E). The levels of beige adipocyte markers, including *TBX1*, *SHOX2*, *TMEM26*, and *CD137* were relatively stable from days 14 to 30, whereas expression of white adipocyte markers gradually increased over time (Figure 1E). These results indicate that goat PRAT exhibits BAT characteristics at postnatal day 1 and gradually undergoes involution into WAT by day 90.

We next investigated potential developmental patterns in gene expression at different ages and identified five major clusters comprising 4454 genes using the maSigPro‐GLM algorithm [32] (Figure 1F). Among these, 1295 genes (776 and 519 genes in clusters 1 and 2, respectively) were typically downregulated during PRAT whitening (Figure 1F). Functional enrichment analysis revealed that these genes are mainly associated with mitochondrial function and energy metabolism, including processes such as mitochondrion organization, mitochondrial transport, tricarboxylic acid cycle, and fatty acid metabolic process (Figure 1G). Conversely, 3159 genes (742, 2046, and 371 genes in clusters 3, 4, and 5, respectively) were typically upregulated during PRAT whitening (Figure 1F). These genes were primarily involved in immune regulation and lipid metabolism, including pathways such as positive regulation of immune response, immune effector process, and membrane lipid metabolic process (Figure 1G). These results suggest that PRAT whitening involves a metabolic shift from mitochondrial oxidative metabolism to immune regulation and lipid remodeling.

### 2.2 The Active Chromatin Landscape Changes During PRAT Whitening in Goats

Transcriptional changes are associated with alterations in the epigenetic landscape at cell‐type‐specific promoters and enhancers [33, 34]. To explore active histone modifications changes associated with PRAT whitening, we mapped the genomic distribution of H3K4me3 and H3K27ac histone modifications (*n* = 2 biological replicates) in BAT (day 1) and WAT (day 90). In total, 8 ChIP‐seq libraries were sequenced, yielding 341.3 million effective reads, with an average of 42.7 million reads per library (Table S1). A high correlation was observed between biological replicates for both H3K4me3 and H3K27ac in BAT and WAT (Figure S2A). Of note, these histone modification marks were significantly enriched around transcription start sites (TSSs) (Figure S2B). As expected, H3K4me3 signals were predominantly enriched at promoter regions, whereas H3K27ac signals were mainly localized to distal intergenic regulatory regions (Figure S2C). These results indicate that the H3K4me3 and H3K27ac ChIP‐seq libraries are high‐quality and reliable for downstream analyses.

**FIGURE 2:**
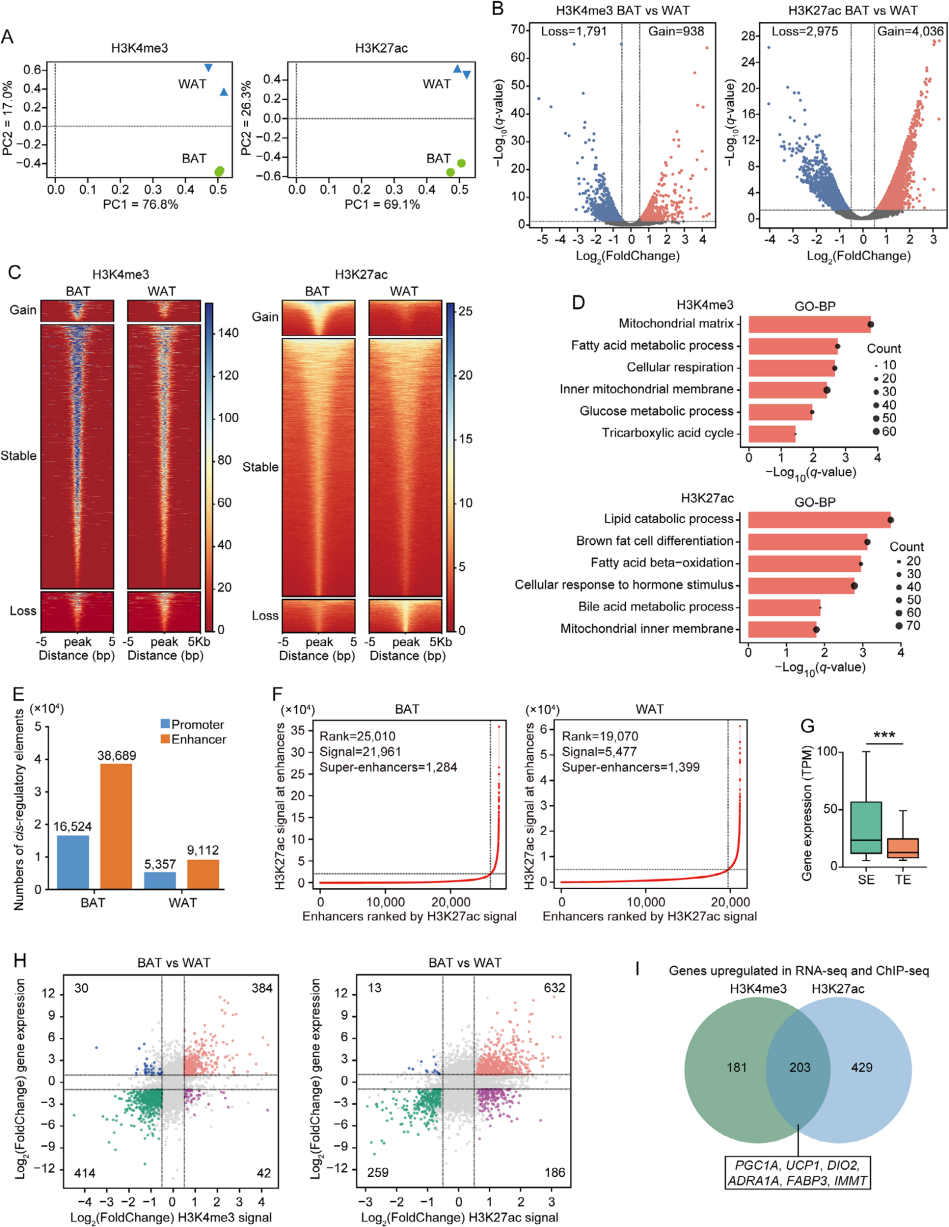
PRAT whitening is associated with global changes in the active chromatin landscape. (A) PCA plots of H3K4me3 and H3K27ac ChIP‐seq profiles in BAT and WAT. (B) Volcano plots of differentially enriched H3K4me3 and H3K27ac ChIP‐seq peaks (|log_2_ fold change| > 0.58, FDR < 0.05) between BAT and WAT. (C) Heatmaps showing changes in H3K4me3 and H3K27ac ChIP‐seq peaks (gained, stable, or loss) between BAT and WAT. (D) GO functional enrichment analysis of genes associated with gained H3K4me3 and H3K27ac peaks. Count represents the number of genes annotated from ChIP‐seq differential peaks that are enriched in the biological process. (E) Number of *cis*‐regulatory elements (promoters and enhancers) identified in brown adipose tissue (BAT) and white adipose tissue (WAT). (F) Distribution of H3K27ac ChIP‐seq signals across enhancers and super‐enhancers in BAT and WAT. (G) Statistical comparison and representative expression profiles of genes associated with super‐enhancers. SE and TE indicate super‐ and typical enhancers, respectively. *p* values were calculated using a two‐sided unpaired Wilcoxon test. *** *p* < 0.001. (H) Scatter plots showing the relationship between gene expression changes and histone mark levels (H3K4me3, left; H3K27ac, right) in BAT compared to WAT. Colored dots represent genes with coordinated upregulation (pink) or downregulation (green), with gene counts indicated in each quadrant. (I) Venn diagram showing overlap between genes upregulated at both the transcriptional and chromatin levels (H3K4me3 and H3K27ac) in BAT compared to WAT.

PCA revealed that the samples clustered into two distinct groups for each histone modification mark (Figure 2A), indicating different patterns of histone modifications (H3K4me3 and H3K27ac) between BAT and WAT. To further investigate the changes in H3K4me3 and H3K27ac signals, the differences between BAT and WAT were calculated (Table S3). The analysis revealed that 2729 signals exhibited differences in H3K4me3 between BAT and WAT, while 7011 differential peaks were identified in H3K27ac between BAT and WAT (Figure 2B,C). Moreover, genes associated with differential H3K4me3 signals between BAT and WAT were enriched in Gene Ontology (GO) terms related to the mitochondrial matrix, fatty acid metabolic process, cellular respiration, inner mitochondrial membrane, glucose metabolic process, and tricarboxylic acid cycle (Figure 2D), while genes associated with differential H3K27ac signals were enriched in GO terms associated with lipid catabolic process, brown fat cell differentiation, fatty acid beta‐oxidation, cellular response to hormone stimulus, bile acid metabolic process, and mitochondrial inner membrane (Figure 2D). In addition, 69682 non‐redundant *cis*‐regulatory elements were identified in BAT and WAT, including 21,881 promoters and 47,801 enhancers (Figure 2E, Table S4). Super‐enhancers (SEs) have been shown to play critical regulatory roles in various cells and tissues, with H3K27ac serving as a classical marker for SEs [35, 36]. We identified 1284 and 1399 SEs in BAT and WAT, respectively, using the ROSE algorithm [36, 37] (Figure 2F; Table S5). As expected, SE‐associated genes displayed significantly higher expression levels than those associated with typical enhancers (Figure 2G).

To identify candidate genes involved in maintaining the browning capacity of PRAT, we examined histone modifications and gene expression levels during PRAT whitening. Our analysis revealed that 384 genes were preferentially upregulated, exhibiting increased transcript levels and H3K4me3 enrichment in BAT. Similarly, 632 genes showed preferential upregulation with increased transcript levels and H3K27ac enrichment in BAT (Figure 2H). Conversely, 414 genes were preferentially downregulated, accompanied by reduced transcript levels and H3K4me3 enrichment in BAT, while 259 genes showed preferential downregulation and exhibited decreased transcript levels and H3K27ac enrichment in BAT (Figure 2H). We then focused on known brown and white adipocyte associated genes. Among the genes that were upregulated and exhibited common gain in H3K4me3 and H3K27ac in BAT were those involved in thermogenesis and mitochondrial function, including *PGC1A*, *UCP1*, *DIO2*, *ADRA1A*, *FABP3*, and *IMMT* (Figure 2I). In contrast, genes that were downregulated and showed common loss in H3K4me3 and H3K27ac in BAT were associated with BAT whitening and inflammatory responses, including *ADRA2A*, *CASP8*, *PTGS2*, *TLR2*, and *EPSTI1* (Figure S2D). These findings suggest that changes in H3K4me3 and H3K27ac patterns during PRAT whitening affect the expression of genes critical for maintaining brown adipocyte identity and function, thereby contributing to the gradual transition from BAT to WAT.

### 2.3 The Chromatin Accessibility Landscape Changes During PRAT Whitening in Goats

Changes in gene expression are often accompanied by alterations in chromatin organization and chromatin accessibility [38]. To investigate chromatin accessibility changes associated with PRAT whitening, we performed a genome‐wide ATAC‐seq (*n* = 3 biological replicates) in BAT and WAT. In total, 6 ATAC‐seq libraries were sequenced, generating 520.5 million effective reads, with an average of 86.8 million reads per library (Table S1). The insert size distribution of each ATAC‐seq library exhibited clear nucleosomal periodicity (Figure S3A). Moreover, the chromatin accessibility profiles showed a relatively good correlation between the three biological replicates (Figure S3B). Of note, ATAC‐seq signals were significantly enriched around TSSs (Figure S3C). As expected, ATAC‐seq signals were preferentially enriched in promoter and distal intergenic regulatory regions (Figure S3D). These results indicate that our data exhibit high reproducibility and reliability in assessing chromatin accessibility.

**FIGURE 3:**
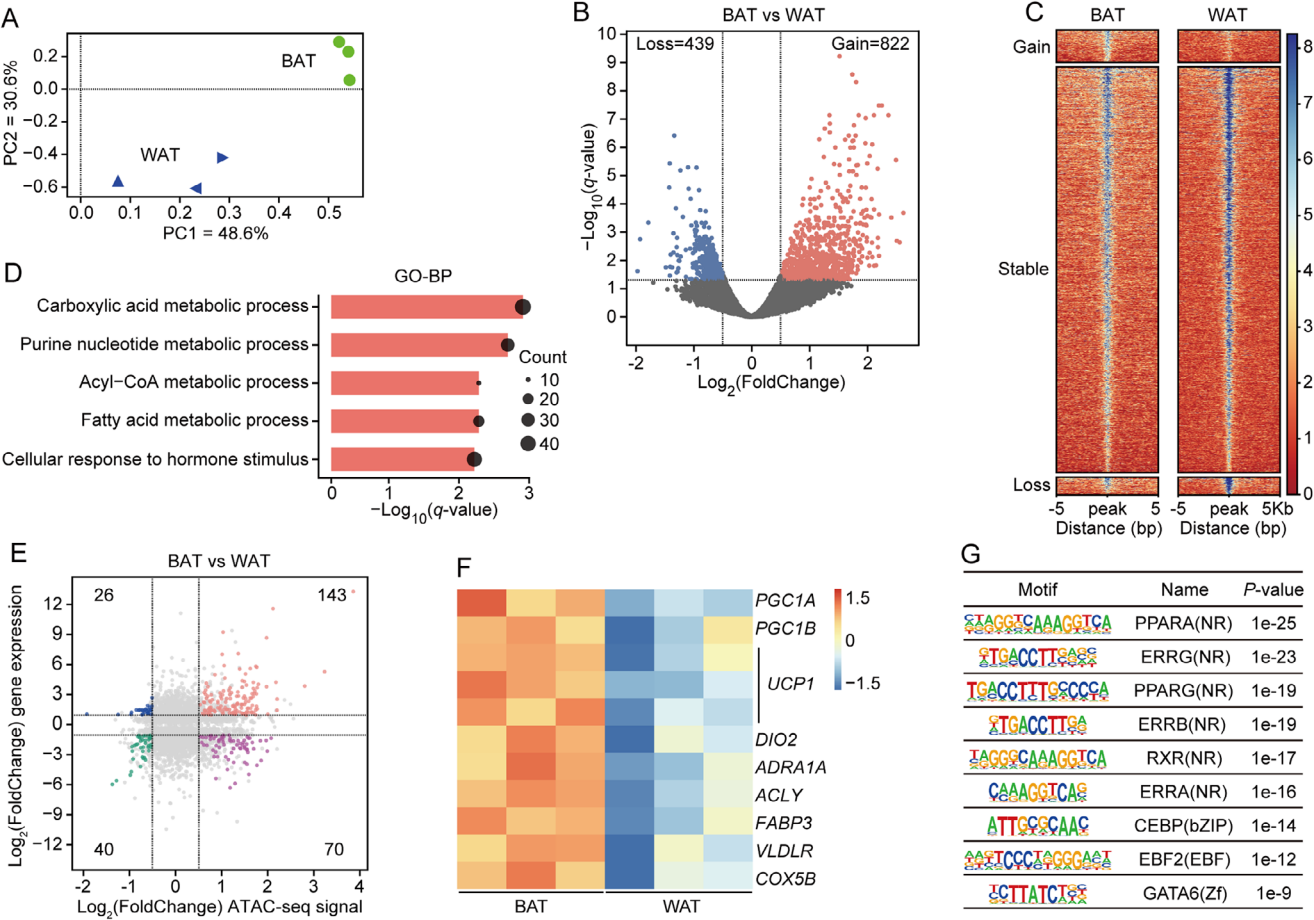
PRAT whitening is associated with global changes in chromatin accessibility. (A) PCA plots of ATAC‐seq profiles in BAT and WAT. (B) Volcano plot of differentially accessible ATAC‐seq peaks (|log_2_ fold change| > 0.58, FDR < 0.05) between BAT and WAT. (C) Heatmaps showing changes in ATAC‐seq peaks (gained, stable, or loss) between BAT and WAT. (D) GO functional enrichment analysis of genes associated with gained ATAC‐seq peaks. Count represents the number of genes annotated from ATAC‐seq differential peaks that are enriched in the biological process. (E) Scatter plots showing the relationship between gene expression changes and chromatin accessibility levels in BAT compared to WAT. Colored dots represent genes with coordinated upregulation (pink) or downregulation (green), with gene counts indicated in each quadrant. (F) Heatmap showing ATAC‐seq signals of thermogenesis and mitochondrial function related genes in BAT and WAT. Multiple rows for a gene (e.g., *UCP1*) denote distinct chromatin accessibility peaks located at different regulatory regions within the gene locus. (G) Known TF motif analysis of gained ATAC‐seq peaks.

To explore genome‐wide changes in chromatin accessibility landscape between BAT and WAT, PCA revealed distinct patterns of global changes in chromatin accessibility (Figure 3A). Subsequently, differential chromatin accessibility analysis of ATAC‐seq peaks between BAT and WAT was performed using the DEseq2 package (Table S6). We identified 822 regions with increased chromatin accessibility and 439 regions with decreased chromatin accessibility (Figure 3B,C). Furthermore, genes associated with the differentially accessible peaks were annotated to significantly enriched GO terms, including carboxylic acid metabolic process, purine nucleotide metabolic process, fatty acid metabolic process, acyl‐CoA metabolic process, and cellular response to hormone stimulus (Figure 3D).

We then performed an integrative analysis of ATAC‐seq and RNA‐seq datasets, identifying 143 genes that were preferentially upregulated, with increased transcript levels and chromatin accessibility in BAT (Figure 3E). Conversely, 40 genes were preferentially downregulated, exhibiting reduced transcript levels and chromatin accessibility in BAT (Figure 3E). Among the genes that were upregulated and exhibited increased chromatin accessibility in BAT were those involved in thermogenesis and mitochondrial function, including *PGC1A*, *PGC1B*, *UCP1*, *DIO2*, *ADRA1A*, *ACLY*, *FABP3*, *VLDLR*, and *COX5B* (Figure 3F). In contrast, genes that were downregulated and showed decreased chromatin accessibility in BAT were associated with BAT whitening and inflammatory responses, including *TLE3* and *GRN*. To identify the key factors that potentially promote chromatin accessibility in BAT, we performed TF motif enrichment analysis for the ATAC‐seq gained peaks. Our analysis revealed significant enrichment of several TF‐binding sites, including the PPAR family (PPARA and PPARG), the ERR family (ERRA, ERRB, and ERRG), RXR, CEBP, EBF2, and GATA6 in differentially accessible ATAC‐seq peaks (Figure 3G). These results indicate that significant alterations in chromatin accessibility during PRAT whitening reduce accessibility at key regulators of brown adipocyte identity and thermogenesis, thereby driving the conversion of BAT to WAT.

### 2.4 Identification and Characterization of PGC1A Active Enhancers

**FIGURE 4:**
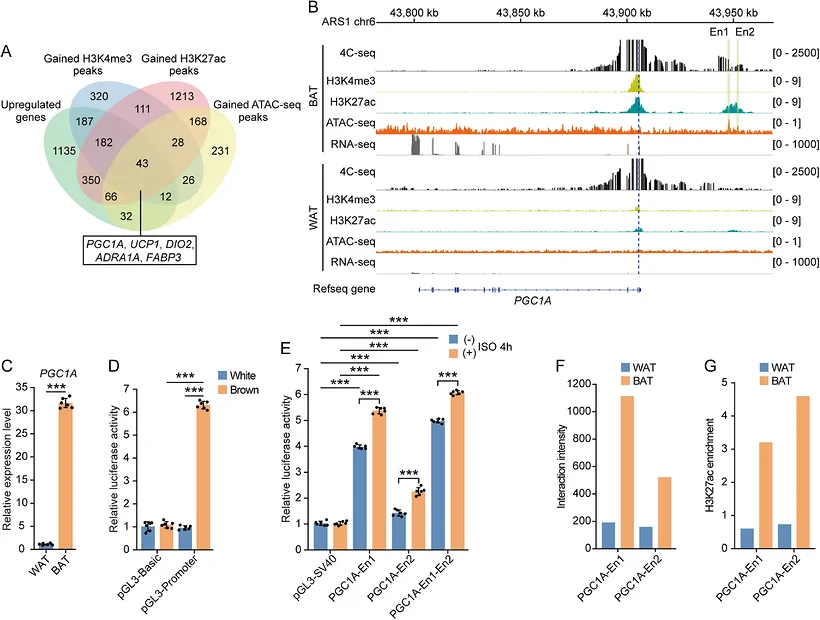
Identification of *PGC1A* active enhancers in BAT and WAT. (A) Venn diagram showing the overlap of DEGs identified by RNA‐seq and genes associated with differentially regulated peaks identified by ChIP‐seq and ATAC‐seq. (B) Integrative Genomics Viewer (IGV) screenshot showing putative active enhancers of *PGC1A* in BAT and WAT. Upper track shows circularized chromosome conformation capture followed by sequencing (4C‐seq) interaction profiles of *PGC1A*. The blue dotted line represents the viewpoint of *PGC1A*. Lower tracks show ChIP‐seq (including H3K4me3 and H3K27ac), ATAC‐seq, and RNA‐seq datasets at the *PGC1A* loci in BAT and WAT. The green column represents putative active enhancers. (C) qRT‐PCR analysis of *PGC1A* expression in BAT and WAT (*n* = 6). (D) Luciferase reporter assay assessing *PGC1A* promoter activity in goat brown and white adipocytes. pGL3‐Basic (empty vector) or pGL3‐Promoter carrying a 2500 bp fragment (chr6:43,904,684‐43,907,183) from the *PGC1A* promoter region (*n* = 6). E) Luciferase reporter assays assessing putative active enhancers of *PGC1A* in goat brown adipocytes. On day 8 of differentiation, cells were treated with 10 µM isoproterenol (ISO) or vehicle for 4 h before luciferase assay (*n* = 6). (F) Signal values for average chromatin interaction intensity at active enhancer regions in BAT and WAT were obtained from bigwig files on IGV. Interaction intensity indicates the normalized frequency of chromatin contacts between the *PGC1A* promoter (viewpoint) and distal genomic regions. (G) Signal values for average H3K27ac histone enrichment at active enhancer regions in BAT and WAT were obtained from bigwig files on IGV. Data are presented as mean ± SEM. For statistical analysis, two‐tailed unpaired Student's *t*‐tests were performed in C; one‐way ANOVA plus Tukey's post hoc tests were performed in D and E. *** *p* < 0.001.

We hypothesized that genes promoting adipocyte thermogenesis would exhibit increased expression levels, active chromatin states, and chromatin accessibility in BAT compared to WAT. To identify candidate genes involved in the thermogenic function of BAT, we integrated upregulated genes with those associated with differentially enriched peaks identified by ChIP‐seq and ATAC‐seq (Figure 4A). This analysis identified a cluster of 43 overlapping genes as candidates associated with brown adipocyte thermogenesis. Among them, *PGC1A*, *UCP1*, *DIO2*, *ADRA1A*, and *FABP3* were directly involved in mitochondrial function and thermogenic processes in brown fat (Figure 4A). We focused on the master regulator PGC1A, a key mediator of WAT browning and metabolic homeostasis [39, 40]. Notably, the H3K4me3 signal was significantly enriched at the *PGC1A* promoter in BAT, where its transcription level was higher compared to WAT (Figure 4B). We further examined the transcription levels of *PGC1A* in BAT and WAT (Figure 4C), both patterns matched the dynamics of chromatin states and chromatin accessibility. Consistently, luciferase reporter assays using pGL3‐Basic containing a 2500 bp fragment of the *PGC1A* promoter region showed a significant increase in luciferase activity compared to the empty vector in goat brown adipocytes, but not in goat white adipocytes (Figure 4D).

To investigate chromatin interactions of *PGC1A* in BAT and WAT, we performed 4C‐seq assays (*n* = 2 biological replicates) using the *PGC1A* promoter as the viewpoint (Figure S4A). A total of 137.3 million filtered reads were obtained, averaging 34.3 million reads per 4C dataset (Table S7). Among these, 61.48% to 67.58% of reads in the four datasets distributed on the *cis*‐chromosome (Figure S4B). All datasets conformed to the ‘*cis*/overall ratio of >40%’ criterion, indicating high data quality [41]. To identify active enhancers regulating *PGC1A*, we integrated 4C‐seq, ChIP‐seq, ATAC‐seq, and RNA‐seq datasets from BAT and WAT. We identified two tissue‐specific putative active enhancers in BAT, these regions showed significant chromatin interactions with the *PGC1A* promoter and overlapped with both H3K27ac and ATAC‐seq signals (Figure 4B).

To assess the enhancer activity of *PGC1A*, one or both of the two putative enhancer regions were cloned into the pGL3‐SV40 luciferase vector and transfected into goat brown adipocytes. The results demonstrated that PGC1A‐En1 induced a robust 4.0‐fold increase in luciferase activity compared to the empty vector, whereas PGC1A‐En2 only exhibited a modest 1.4‐fold increase (Figure 4E). Notably, the combination of both PGC1A‐En1 and PGC1A‐En2 (PGC1A‐En1‐En2) led to a 5.0‐fold increase in luciferase activity (Figure 4E). Furthermore, β‐adrenergic stimulation with isoproterenol (ISO) further elevated the luciferase activity of these constructs (Figure 4E). These results suggest that the *PGC1A* enhancers have an additive effect and further activated by cold stimulation.

We next analyzed differences in chromatin interactions and H3K27ac histone modifications at two active enhancers between BAT and WAT. Interaction intensity between the *PGC1A* promoter and these enhancers was significantly higher in BAT, where *PGC1A* is more highly transcribed, compared to WAT (Figure 4F). In addition, these active enhancers exhibited significantly higher levels of H3K27ac histone modifications in BAT than in WAT (Figure 4G). These findings indicate that chromatin interactions and histone modifications may play critical roles in the activity of these enhancers, potentially regulating *PGC1A* expression in brown fat.

### 2.5 PGC1A‐En1 and PGC1A‐En2 Regulate PGC1A Expression and Affect Thermogenesis in Goat Brown Adipocytes

To investigate whether the identified active enhancers regulate *PGC1A* expression in goat brown adipocytes, we first assessed *PGC1A* expression during adipogenic differentiation of stromal vascular fraction (SVF) cells isolated from BAT. *PGC1A* expression increased by day 5 of differentiation and remained elevated through day 8 (Figure S5A). We next evaluated whether the CRISPR‐dCas9‐KRAB interference (CRISPRi) system could be transiently transfected into goat SVF cells and expressed effectively. The qRT‐PCR analysis confirmed robust expression of the CRISPRi system (Figure S5B). Meanwhile, in goat mature brown adipocytes transfected with the CRISPRi system, ISO treatment induced significant upregulation of key thermogenic genes, including *PGC1A*, *UCP1*, and *DIO2* (Figure S5C). These results demonstrate that the CRISPRi system functions effectively in goat brown adipocytes and that β‐adrenergic receptor agonist treatment activates their thermogenic activity.

**FIGURE 5:**
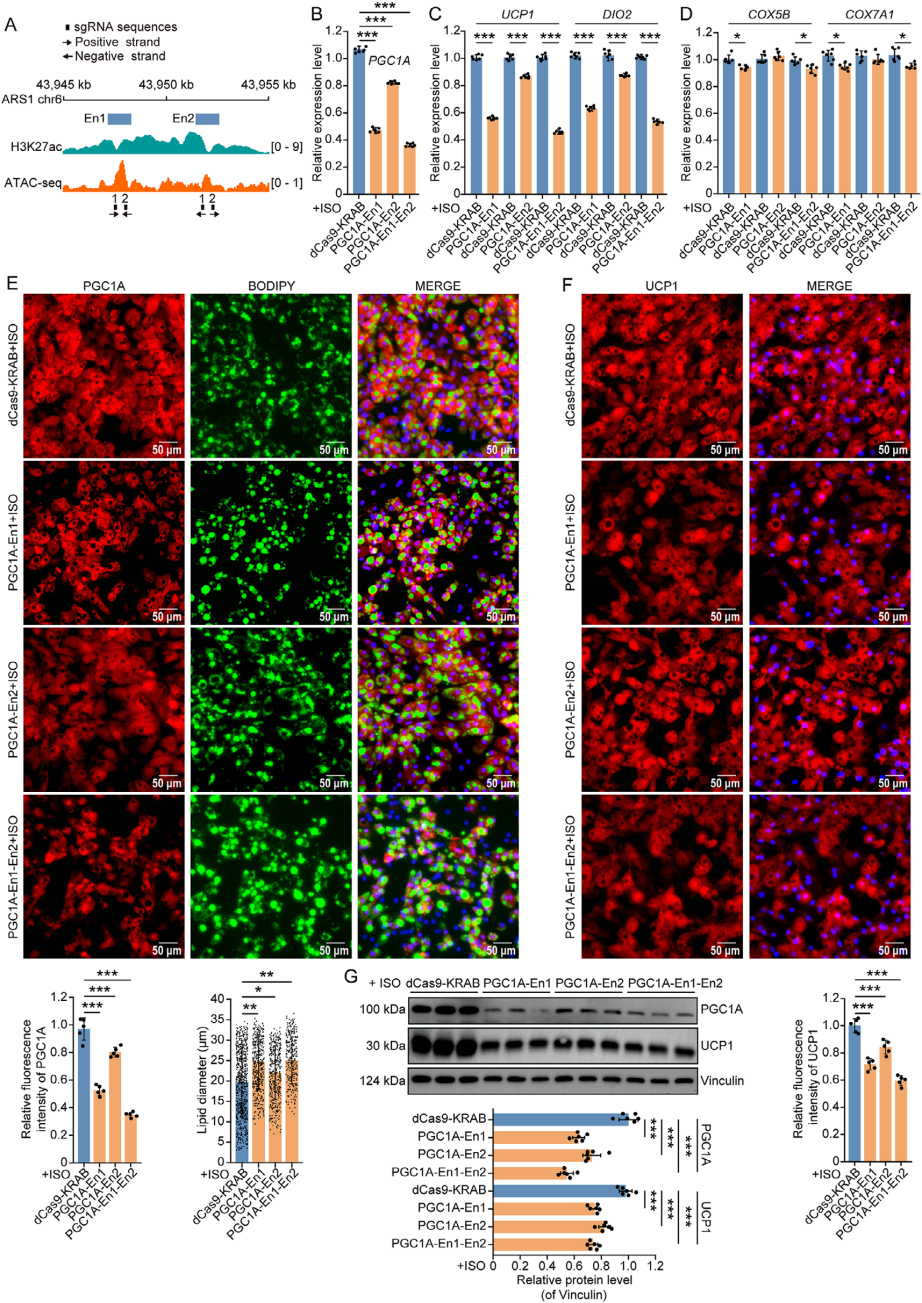
Identification of functional enhancers regulating *PGC1A* expression and affecting thermogenesis. (A) Schematic of dCas9‐KRAB sgRNA target sites at PGC1A‐En1 and PGC1A‐En2. (B–D) qRT‐PCR analysis of *PGC1A*, *UCP1*, *DIO2*, *COX5B*, and *COX7A1* expression in goat brown adipocytes after ISO treatment (*n* = 6). (E) Immunofluorescence staining of PGC1A (red), BODIPY (green), and nuclei (DAPI in blue) in goat brown adipocytes after ISO treatment. Lipid droplet size was measured as the diameter of individual lipid droplets. Scale bar = 50 µm. (F) Immunofluorescence staining of UCP1 (red) and nuclei (DAPI in blue) in goat brown adipocytes after ISO treatment. Scale bar = 50 µm. (G) Western blot analysis of PGC1A and UCP1 in goat brown adipocytes after ISO treatment (*n* = 6). Gray values of protein bands were quantified using ImageJ software, with Vinculin as a normalizer. Data are presented as mean ± SEM. For statistical analysis, one‐way ANOVA plus Tukey's post hoc tests were performed in (B–G). * *p* < 0.05, ** *p* < 0.01, *** *p* < 0.001.

Subsequently, we utilized the CRISPRi system to investigate the regulatory effects of PGC1A‐En1, PGC1A‐En2, and PGC1A‐En1‐En2, respectively, on *PGC1A* transcription (Figure 5A). On day 5 of differentiation, cells were transfected with plasmids expressing dCas9‐KRAB with either control sgRNAs, sgRNAs targeting PGC1A‐En1, sgRNAs targeting PGC1A‐En2, or sgRNAs targeting PGC1A‐En1‐En2. By day 8 of differentiation, CRISPRi‐mediated repression of PGC1A‐En1 or PGC1A‐En1‐En2 led to significant reductions in *PGC1A* expression by 13% and 24%, respectively, compared to the control (Figure S5D). Targeting PGC1A‐En2 did not result in a statistically significant change in *PGC1A* expression (Figure S5D). Notably, in ISO‐treated goat brown adipocytes, repression of PGC1A‐En1, PGC1A‐En2, or PGC1A‐En1‐En2 resulted in more pronounced reductions in *PGC1A* expression—by 53%, 17%, and 64%, respectively (Figure 5B). Moreover, inhibition of PGC1A‐En1 or PGC1A‐En1‐En2 significantly decreased the expression of key thermogenic genes (*UCP1* and *DIO2*) and mitochondrial genes (*COX5B* and *COX7A1*), but only under ISO treatment (Figure 5C,D, Figure S5E,F). Consistently, immunofluorescence staining showed that repression of PGC1A‐En1 or PGC1A‐En1‐En2 markedly reduced PGC1A and UCP1 expression, accompanied by the presence of larger lipid droplets in ISO‐treated goat brown adipocytes (Figure 5E,F), a characteristic commonly associated with impaired brown adipocyte thermogenesis. In addition, Western blot analysis confirmed decreased PGC1A and UCP1 protein levels following repression of PGC1A‐En1 or PGC1A‐En1‐En2 under ISO treatment compared with the control (Figure 5G). Moreover, qPCR analysis showed that repression of PGC1A‐En1, PGC1A‐En2, or PGC1A‐En1‐En2 did not significantly affect mitochondrial DNA (mtDNA) content without ISO stimulation (Figure S5G). However, following ISO treatment, repression of PGC1A‐En1 or PGC1A‐En1‐En2 led to a significant reduction in mtDNA content compared with control cells (Figure S5H). These results demonstrate that PGC1A‐En1 and PGC1A‐En2 control *PGC1A* expression and play critical roles in regulating thermogenesis in goat brown adipocytes.

### 2.6 Repression of Pgc1a‐En1 Reduces Pgc1a Expression and Impairs BAT Thermogenic Capacity in Mice

**FIGURE 6:**
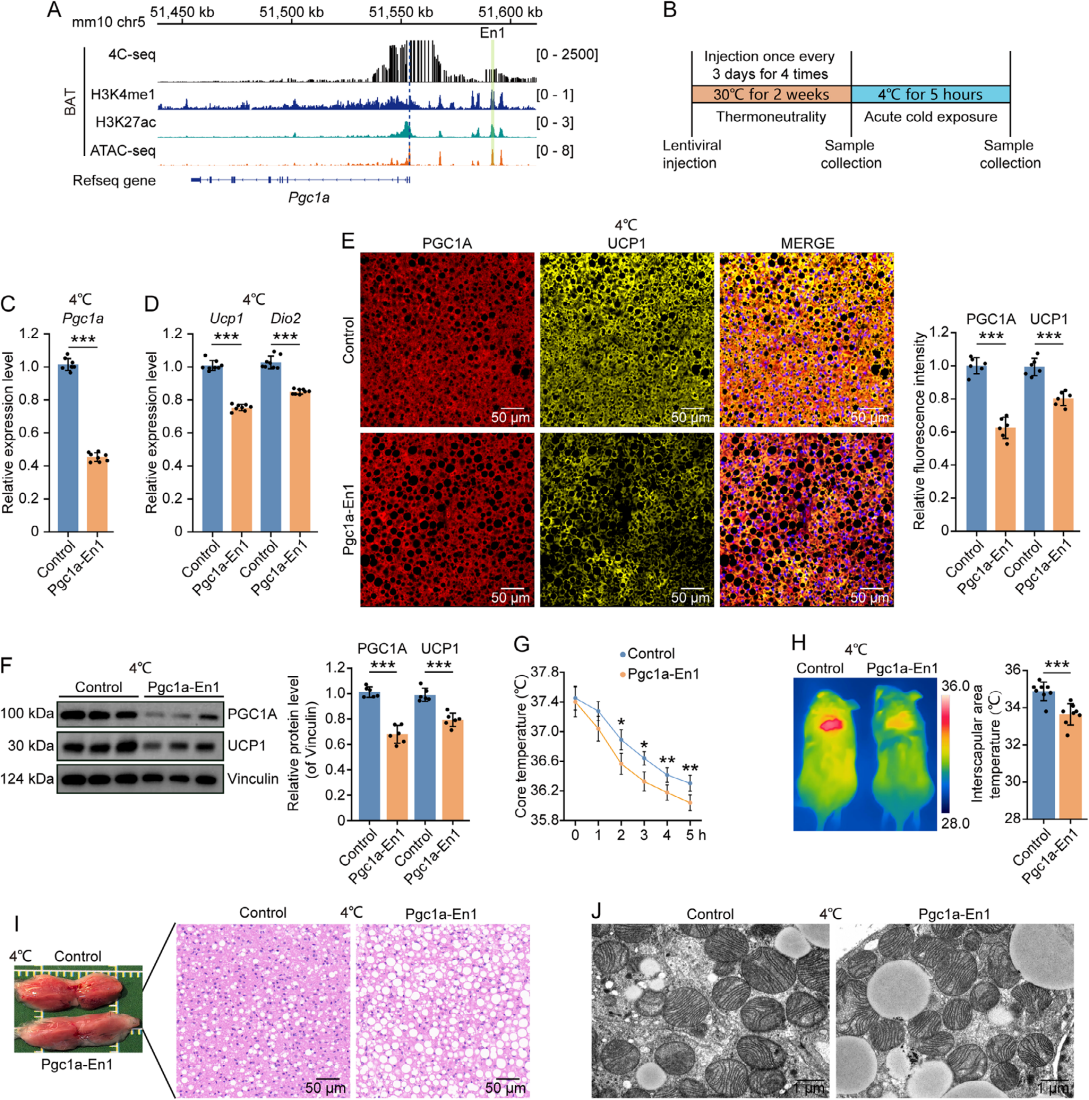
Repression of Pgc1a‐En1 impairs BAT thermogenic capacity in mice under acute cold exposure. (A) Chromatin interaction between the *Pgc1a* promoter and Pgc1a‐En1 in interscapular brown adipose tissue (iBAT). Upper track shows the 4C‐seq interaction profile of *Pgc1a* in iBAT. The blue dotted line represents the viewpoint of *Pgc1a*. Lower tracks show ChIP‐seq (including H3K4me1 and H3K27ac), and ATAC‐seq signals at the *Pgc1a* locus in iBAT. The green column represents putative active enhancer. (B) Schematic of the experimental design for the local injection study. (C,D) qRT‐PCR analysis of *Pgc1a*, *Ucp1*, and *Dio2* expression in iBAT after acute cold exposure (*n* = 8). (E) Immunofluorescence staining of iBAT for PGC1A (red), UCP1 (yellow), and nuclei (blue) after acute cold exposure. Scale bar = 50 µm. (F) Western blot analysis of PGC1A and UCP1 in iBAT after acute cold exposure (*n* = 6). Gray values of protein bands were quantified using ImageJ software, with Vinculin as a normalizer. (G) Core body temperature of mice exposed to cold (4°C). (H) Representative images of the dorsal skin (left) and quantification of average dorsal skin temperature (right) after acute cold exposure (*n* = 8). (I) Representative images of iBAT after acute cold exposure (left). Representative H&E staining of iBAT after acute cold exposure (right). Scale bar = 50 µm. (J) Transmission electron microscopy images of iBAT after acute cold exposure. Scale bar = 1 µm. Data are presented as mean ± SEM. For statistical analysis, two‐tailed unpaired Student's *t*‐tests were performed in (C–H). * *p* < 0.05, ** *p* < 0.01, *** *p* < 0.001.

Although PGC1A‐En1 and PGC1A‐En2 can regulate target gene expression in an additive manner, PGC1A‐En1 appears to act as the central regulatory element. To further investigate the role of PGC1A‐En1 in BAT in vivo, we examined its conservation at three levels (sequence, synteny, and epigenomic conservation) compared to the mouse genome. First, goat coordinates (ARS1) were converted to orthologous mouse (mm10) regions using the UCSC LiftOver tool [42] with the minMatch parameter set to 0.9 (90% sequence identity). Next, to determine whether the conserved mouse Pgc1a‐En1 sequence physically interacts with the *Pgc1a* promoter, we performed 4C‐seq (Figure S6A,B) on interscapular brown adipose tissue (iBAT) and analyzed publicly available datasets, including enhancer‐associated ChIP‐seq (H3K27ac and H3K4me1) and ATAC‐seq data. We observed strong chromatin interactions between the *Pgc1a* promoter and the Pgc1a‐En1 region (Figure 6A). Moreover, the Pgc1a‐En1 region was enriched for H3K27ac and H3K4me1 and exhibited high chromatin accessibility (Figure 6A). These results indicate that Pgc1a‐En1 is not only a conserved sequence in the mouse genome, but also maintains conserved target gene regulatory interactions and epigenomic signatures [43], highlighting its evolutionary importance in regulating *Pgc1a* expression across species.

To investigate the role of Pgc1a‐En1 in BAT function in vivo, we injected the CRISPRi‐mediated repression system targeting Pgc1a‐En1 directly into the iBAT. The experimental design for lentiviral injection is illustrated in Figure 6B. At two weeks after injection, immunofluorescence analysis revealed widespread GFP expression throughout the iBAT (Figure S6C), confirming successful and stable lentiviral infection. The expression of *Pgc1a* in iBAT was evaluated in control mice (injected with dCas9‐KRAB‐GFP lentivirus) and Pgc1a‐En1 mice (injected with dCas9‐KRAB‐Pgc1a‐En1 lentivirus). The results showed that *Pgc1a* expression was moderately reduced under thermoneutral conditions (Figure S6D) and significantly decreased following acute cold exposure (Figure 6C). In addition, the expression of *Ucp1* and *Dio2* in iBAT was markedly lower in Pgc1a‐En1 mice than in controls after acute cold exposure (Figure 6D, Figure S6E). Consistent with these transcriptional changes, immunofluorescence analysis and Western blotting revealed a significant reduction in PGC1A and UCP1 protein levels in Pgc1a‐En1 mice after acute cold exposure (Figure 6E,F, Figure S6F,G). These results suggest that repression of Pgc1a‐En1 reduces *Pgc1a* expression in vivo.

No significant differences were observed in body weight or core temperature between Pgc1a‐En1 and control mice under thermoneutral conditions (Figure S6H,I). However, after cold exposure at 4°C for 5 h, Pgc1a‐En1 mice had significantly lower core temperature than control mice (Figure 6G). Similarly, dorsal skin temperature in the interscapular region was significantly lower in Pgc1a‐En1 mice than in controls after acute cold exposure (Figure 6H), but not under thermoneutral conditions (Figure S6J). Moreover, the iBAT of Pgc1a‐En1 mice appeared whitish in color after acute cold exposure compared to controls (Figure 6I). H&E staining further revealed increased lipid droplet size with more unilocular, white‐like adipocytes in Pgc1a‐En1 mice compared to controls after acute cold exposure (Figure 6I), but not under thermoneutral conditions (Figure S6K). Electron microscopy analysis of iBAT showed no significant differences in mitochondrial abundance or morphology between Pgc1a‐En1 and control mice after acute cold exposure (Figure 6J). However, analysis of mtDNA content demonstrated a significant reduction in Pgc1a‐En1 mice after acute cold exposure (Figure S6L,M). These results suggest that Pgc1a‐En1 is critical for regulating BAT thermogenic capacity in response to cold exposure.

### 2.7 ERRA Cooperates with CBP to Regulate the Activity of En1 is Conserved Among Species

**FIGURE 7:**
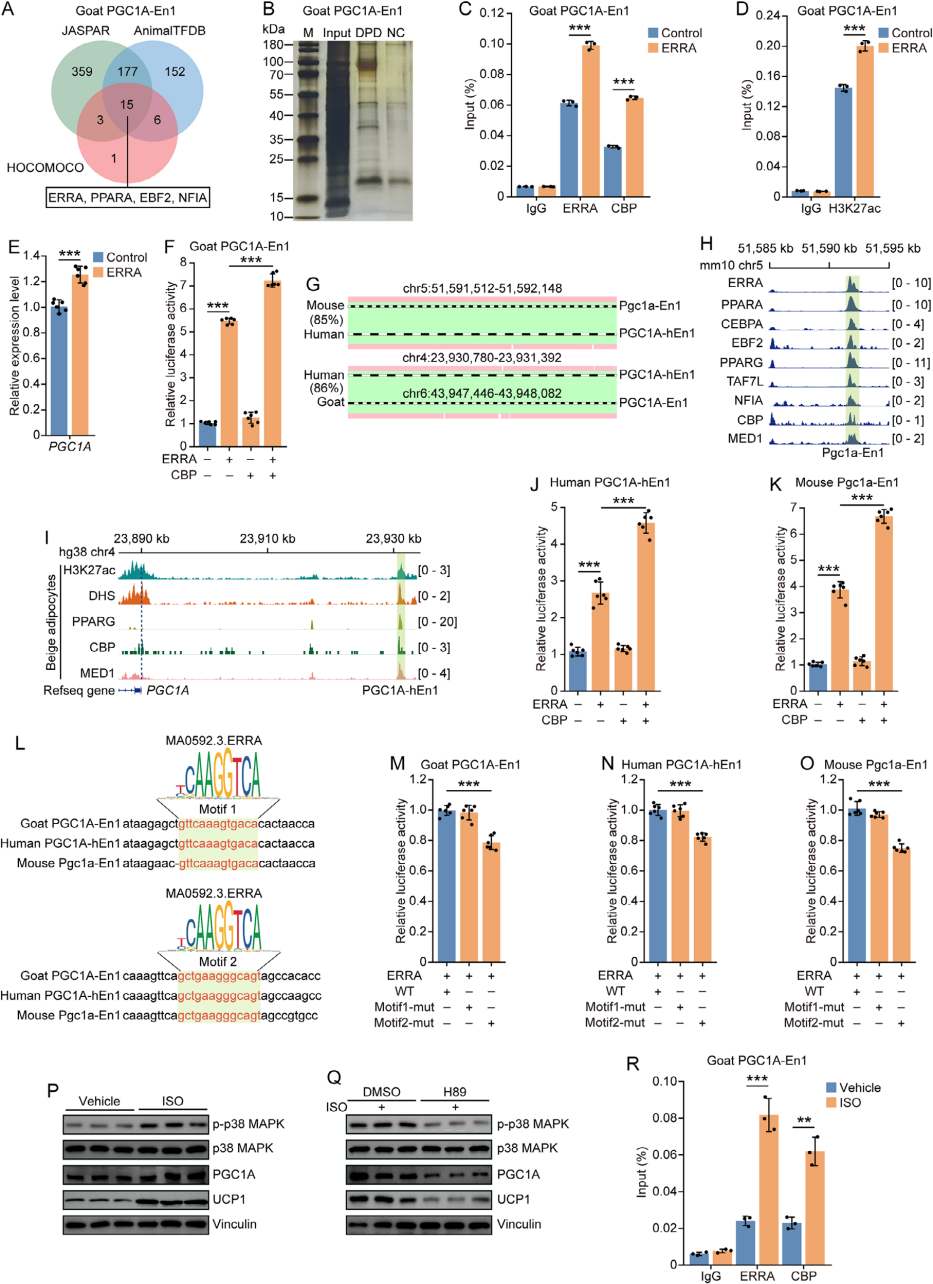
Cooperative regulation of enhancer activity by ERRA and CBP is conserved across species. (A) Venn diagram showing TFs predicted by JASPAR, AnimalTFDB 4.0, and HOCOMOCO. ERRA, PPARA, EBF2, and NFIA were commonly identified by all three databases. (B) Silver staining of nuclear proteins identified by DNA pull‐down (DPD) assay using the goat PGC1A‐En1 probe. M: marker; DPD: PGC1A‐En1 probe group; NC: negative control group. (C, D) ChIP‐qPCR analysis of ERRA, CBP, and H3K27ac enrichment at the PGC1A‐En1 region in goat brown adipocytes (*n* = 3). (E) qRT‐PCR analysis of *PGC1A* expression in goat brown adipocytes (*n* = 6). (F) H293T cells were cotransfected with the goat PGC1A‐En1 construct and expression plasmids for ERRA and/or CBP, either individually or in combination (*n* = 6). (G) Conservation track obtained from the Ensembl Genome Browser with green lines indicating conserved sequences between human and goat, and between human and mouse, respectively. (H) ChIP‐seq profiles showing binding of various TFs at the Pgc1a‐En1 region in mouse brown fat. (I) ChIP‐seq (including H3K27ac, PPARG, CBP, and MED1) and DHS‐seq profiles in beige adipocytes differentiated from human multipotent adipose‐derived stem cells. The green column represents putative active enhancer. (J,K) H293T cells were cotransfected with the human PGC1A‐hEn1 (J) or mouse Pgc1a‐En1 (K) constructs and expression plasmids for ERRA and/or CBP, either individually or in combination (*n* = 6). (L) Detailed alignment of ERRA‐binding sites in goat, human, and mouse. The ERRA motif is shown above. (M–O) Relative luciferase activity of wild‐type (WT) or motif‐mutated enhancers from goat (M), human (N), and mouse (O) cotransfected with the respective ERRA expression constructs (*n* = 6). (P) Western blot analysis of PGC1A, UCP1, Phospho‐p38 MAPK, and total p38 MAPK in goat brown adipocytes after ISO treatment (*n* = 3). (Q) Western blot analysis of PGC1A, UCP1, Phospho‐p38 MAPK, and total p38 MAPK in goat brown adipocytes after ISO treatment in the presence or absence of the PKA inhibitor H89 (*n* = 3). (R) ChIP‐qPCR analysis of ERRA and CBP enrichment at the PGC1A‐En1 region in goat brown adipocytes after ISO treatment (*n* = 3). Data are presented as mean ± SEM. For statistical analysis, two‐tailed unpaired Student's *t*‐tests were performed in E; one‐way ANOVA plus Tukey's post hoc tests were performed in C, D, F, J, K, M–O. ** *p* < 0.01, *** *p* < 0.001.

To investigate the molecular mechanisms controlling the activity of the goat PGC1A‐En1, we performed TF binding site motif analysis using the JASPAR [44], AnimalTFDB 4.0 [45], and HOCOMOCO [46] databases. The results showed that all three databases consistently predicted binding motifs for ERRA, PPARA, EBF2, and NFIA (Figure 7A). Further, DNA pull‐down assay was performed with nuclear extracts from goat BAT using the goat PGC1A‐En1 probe. The binding proteins were separated by SDS‐polyacrylamide gel electrophoresis, and the entire lane was excised and analyzed by mass spectrometry (Figure 7B, Table S8). Consistent with the prediction results of TF binding sites, ERRA, PPARA, and EBF2 were found to bind to the goat PGC1A‐En1 region. These results indicate these TFs as high‐confidence candidates potentially involved in modulating goat PGC1A‐En1 activity.

ERRA is an orphan nuclear receptor involved in mitochondrial biogenesis and adaptive thermogenesis [47]. However, the transcriptional regulatory mechanism through which ERRA regulates *PGC1A* expression remains unclear. Enhancer activation depends on the recruitment of specific TFs and histone‐modifying enzymes [48]. CBP, a histone acetyltransferase, is recruited by numerous TFs to regulate gene expression [33, 49, 50]. Therefore, we speculated that ERRA and CBP synergistically enhance the activity of the goat PGC1A‐En1, thereby promoting the transcriptional activation of *PGC1A*. ChIP‐qPCR analysis revealed that ERRA overexpression markedly increased the binding of both ERRA and CBP to the goat PGC1A‐En1 region (Figure 7C). Moreover, ERRA overexpression significantly elevated H3K27ac enrichment at the goat PGC1A‐En1 region (Figure 7D) and significantly upregulated *PGC1A* mRNA levels (Figure 7E). To further examine the functional significance of ERRA and CBP interaction in activating the goat PGC1A‐En1, we cotransfected ERRA and CBP with the goat PGC1A‐En1 luciferase reporter construct, either individually or in combination. ERRA alone induced a 5.5‐fold increase in luciferase activity, whereas cotransfection with both ERRA and CBP resulted in a more robust 7.2‐fold activation (Figure 7F). These results suggest that ERRA cooperates with CBP at the goat PGC1A‐En1 region to enhance local H3K27ac enrichment, thereby promoting *PGC1A* transcription.

To assess whether the cooperative regulation of enhancer activity by ERRA and CBP is conserved across species, we first examined the evolutionary conservation of the goat PGC1A‐En1 sequence among goat, human, and mouse. We found that the sequence identity between human and goat, and between human and mouse, exceeded 80% (Figure 7G). We then analyzed publicly available ChIP‐seq datasets from mouse brown fat to identify TFs enriched at the Pgc1a‐En1 region. Consistent with TF binding observed at the goat PGC1A‐En1 region, ERRA, PPARA, EBF2, NFIA, and CBP were found to bind to the mouse Pgc1a‐En1 region (Figure 7H). In addition, other key brown fat‐related TFs, including CEBPA, PPARG, TAF7L, and the mediator subunit MED1, also showed significant binding at this locus (Figure 7H). These findings suggest that the mouse Pgc1a‐En1 region plays an important role in regulating *Pgc1a* transcription. Furthermore, epigenetic analysis of human thermogenic adipocytes revealed that the conserved region corresponding to goat PGC1A‐En1 and mouse Pgc1a‐En1, referred to as PGC1A‐hEn1, was markedly enriched for active enhancer features, including H3K27ac and DHS‐seq (chromatin accessibility), as well as PPARG, CBP, and MED1 occupancy (Figure 7I). We next performed luciferase reporter assays to evaluate the cooperative regulation of enhancer activity by ERRA and CBP. H293T cells were transfected with luciferase constructs containing either the human PGC1A‐hEn1 or the mouse Pgc1a‐En1 enhancer, together with their respective ERRA and CBP expression vectors, individually or in combination. Consistent with the goat PGC1A‐En1 results, ERRA alone activated both the mouse Pgc1a‐En1 and human PGC1A‐hEn1, whereas cotransfection with ERRA and CBP further enhanced enhancer activity (Figure 7J,K). These results indicate that ERRA cooperates with CBP to regulate the activity of enhancers is conserved among species.

To identify conserved ERRA‐binding sites that may regulate enhancer activity across species, we performed motif analysis of the goat PGC1A‐En1, mouse Pgc1a‐En1, and human PGC1A‐hEn1 sequences using the JASPAR database. This analysis revealed that these enhancer sequences contain highly conserved ERRA motifs, designated as ERRA‐motif1 and ERRA‐motif2, which are shared among goat, mouse, and human (Figure 7L). We then constructed PGL3‐SV40‐PGC1A‐En1, PGL3‐SV40‐PGC1A‐hEn1, and PGL3‐SV40‐Pgc1a‐En1 plasmids, each carrying individual deletions of the respective ERRA‐binding sites. Luciferase reporter assays were performed in H293T cells cotransfected with the ERRA overexpression plasmid. Mutations in ERRA‐motif2 significantly reduced enhancer activity across species, whereas mutations in ERRA‐motif1 had no significant effect (Figure 7M–O). These results suggest that ERRA directly regulates enhancer activity across species by binding to the ERRA‐motif2 site.

Previous studies have demonstrated that β‐adrenergic signaling activates BAT thermogenesis primarily through the PKA‐p38 MAPK signaling cascade [51, 52]. To investigate whether this pathway links β‐adrenergic stimulation to *PGC1A* enhancer activation, we first examined p38 MAPK signaling in goat brown adipocytes upon ISO treatment. Western blot analysis showed that ISO treatment markedly increased the phosphorylation of p38 MAPK, accompanied by elevated protein levels of PGC1A and UCP1 compared with vehicle‐treated controls (Figure 7P). Subsequently, goat brown adipocytes were treated with ISO in the presence or absence of the PKA inhibitor H89. The result revealed that inhibition of PKA effectively suppressed ISO‐induced p38 MAPK phosphorylation and substantially attenuated the upregulation of PGC1A and UCP1 protein levels (Figure 7Q). Given that ERRA and CBP were identified as key regulators of PGC1A‐En1 activity, we next examined whether β‐adrenergic stimulation promotes their recruitment to the enhancer region. ChIP‐qPCR analysis revealed that ISO treatment significantly increased the enrichment of both ERRA and CBP at the goat PGC1A‐En1 locus compared with controls (Figure 7R). These results suggest that β‐adrenergic stimulation may activate the PGC1A‐En1 enhancer through a PKA‐p38 MAPK‐dependent pathway, facilitating the recruitment of ERRA and CBP to the enhancer region and thereby promoting *PGC1A* transcription.

### 2.8 PGC1A‐hEn1 Regulates PGC1A Expression and Affects Thermogenesis in Human Brown Adipocytes

**FIGURE 8:**
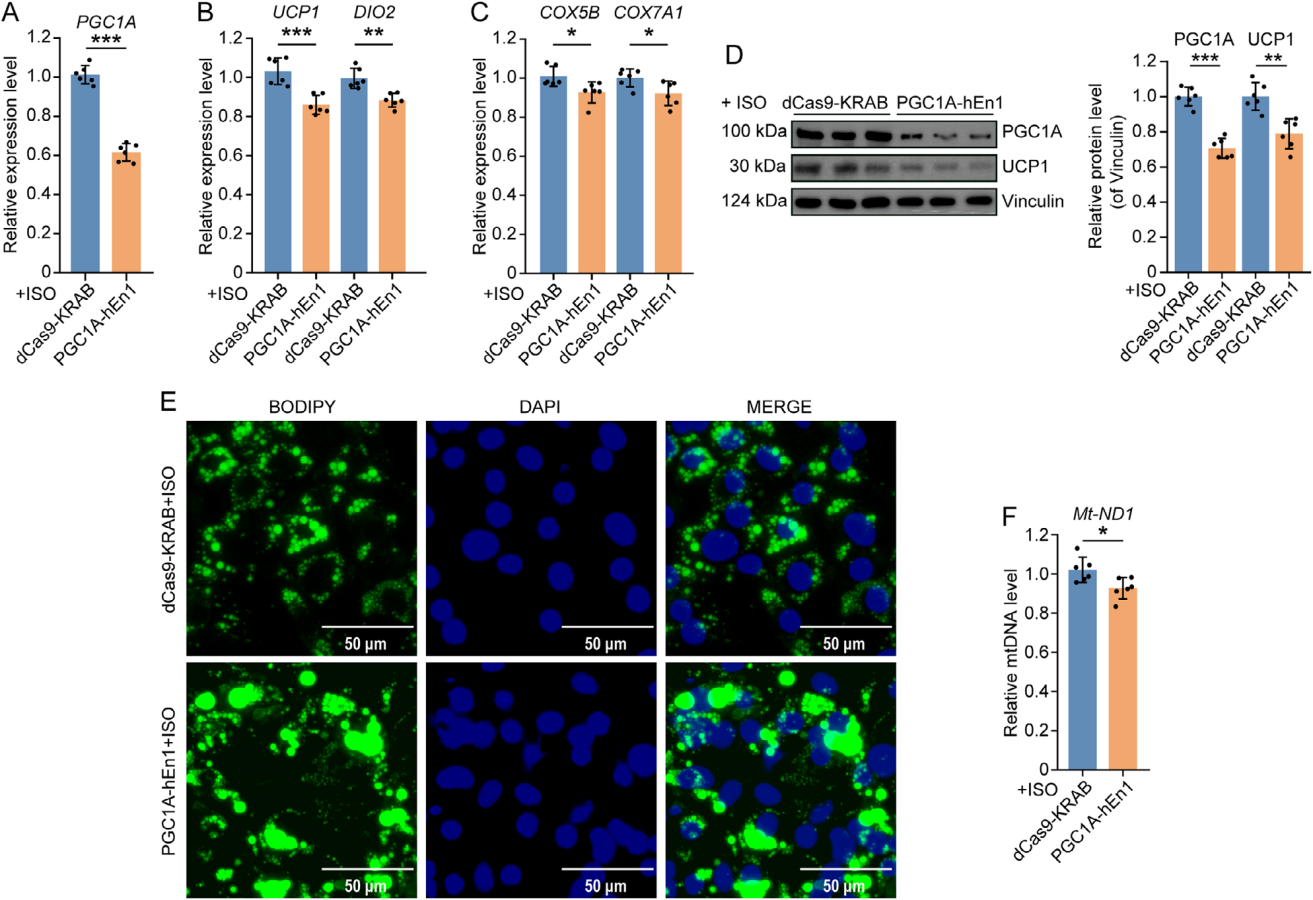
PGC1A‐hEn1 regulates *PGC1A* expression and thermogenic function. (A–C) qRT‐PCR analysis of *PGC1A*, *UCP1*, *DIO2*, *COX5B*, and *COX7A1* expression in human brown adipocytes after ISO treatment (*n* = 6). (D) Western blot analysis of PGC1A and UCP1 in human brown adipocytes after ISO treatment (*n* = 6). Gray values of protein bands were quantified using ImageJ software, with Vinculin as a normalizer. (E) Immunofluorescence staining of BODIPY (green) and nuclei (DAPI in blue) in human brown adipocytes after ISO treatment. Scale bar = 50 µm. (F) qPCR analysis of mtDNA content in human brown adipocytes after ISO treatment (*n* = 6). Data are presented as mean ± SEM. For statistical analysis, two‐tailed unpaired Student's *t*‐tests were performed in (A–D, F). * *p* < 0.05, ** *p* < 0.01, *** *p* < 0.001.

To determine whether the conserved human enhancer PGC1A‐hEn1 regulates *PGC1A* expression in human brown adipocytes, we employed a CRISPRi strategy to specifically repress PGC1A‐hEn1 in brown adipocytes differentiated from the immortalized human brown preadipocytes (hTERT A41hBAT‐SVF [53]). On day 14 of differentiation, cells were transfected with plasmids encoding dCas9‐KRAB together with either control sgRNAs or sgRNAs targeting PGC1A‐hEn1. By day 18, repression of PGC1A‐hEn1 reduced *PGC1A* mRNA levels by approximately 10% compared with the control group (Figure S7A). Notably, upon ISO stimulation, inhibition of PGC1A‐hEn1 led to a more pronounced reduction in *PGC1A* expression, decreasing to approximately 39% of control levels (Figure 8A).

Furthermore, repression of PGC1A‐hEn1 significantly decreased the expression of thermogenic marker genes (*UCP1* and *DIO2*) and mitochondrial genes (*COX5B* and *COX7A1*), an effect observed exclusively under ISO‐treated conditions (Figure 8B,C, Figure S7B, C). Consistently, Western blot analysis revealed marked reductions in PGC1A and UCP1 protein levels following PGC1A‐hEn1 repression, accompanied by the accumulation of enlarged lipid droplets in ISO‐treated human brown adipocytes (Figure 8D,E). Moreover, qPCR analysis demonstrated that repression of PGC1A‐hEn1 significantly reduced mtDNA content compared with control cells under ISO treatment (Figure 8F, Figure S7D). These results demonstrate that the conserved human enhancer PGC1A‐hEn1 functions as a critical regulator of *PGC1A* expression and is required for proper thermogenic activation in human brown adipocytes.

## 3 Discussion

**FIGURE 9:**
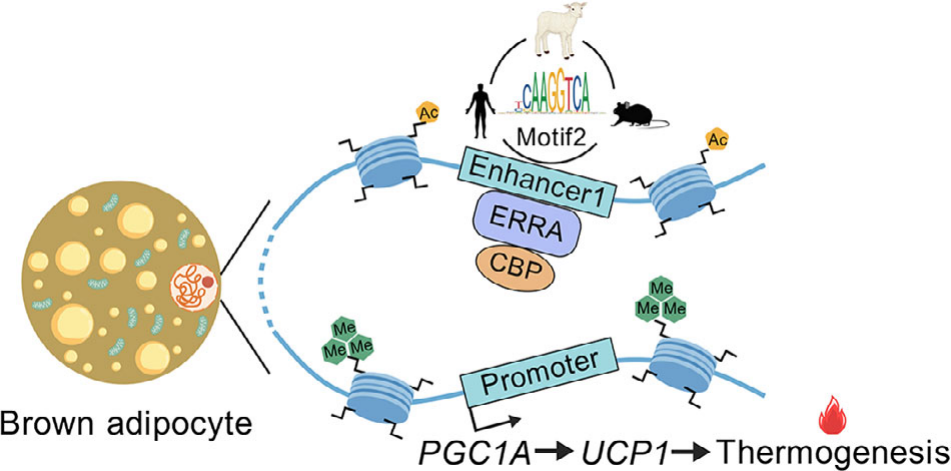
Proposed model illustrating conserved enhancer‐mediated regulation of *PGC1A* expression in brown adipocytes. The conserved enhancer PGC1A‐En1 interacts with the *PGC1A* promoter and requires recruitment of ERRA and the coactivator CBP to promote local H3K27ac enrichment and activate *PGC1A* transcription. Elevated *PGC1A* expression subsequently drives UCP1‐dependent thermogenesis and helps maintain brown adipocyte identity across species. Schematic illustrations were created with BioGDP.com [71].

BAT activation is closely associated with age [54, 55], and no effective strategy has been established to restore functional BAT in aging or obese individuals. Better understanding the underlying regulatory mechanisms of BAT activation may provide future therapeutic targets for obesity and related metabolic diseases. In this study, we performed comprehensively analyses of transcriptional expression, active histone modifications, chromatin accessibility, and *PGC1A* chromatin interactions across a defined time course of PRAT whitening in goats. We identified a functionally conserved enhancer that is critical for *PGC1A* expression and thermogenesis in brown fat (Figure 9).

BAT involution is not unique to humans and has also been reported in large mammals such as goats [15], sheep [56], and rabbits [57]. Our findings indicated that goat PRAT undergoes a progressive whitening process after birth. Histological analysis and UCP1 immunohistochemistry confirmed a BAT phenotype at day 1, which gradually transitioned to a WAT‐like phenotype by day 90. Notably, between days 14 and 30, PRAT exhibited a mixed morphology characterized by both multilocular and unilocular lipid droplets, indicative of a transitional stage. RNA‐seq analysis revealed that PRAT from days 14 and 30 displayed highly similar transcriptomic profiles and only a limited number of DEGs, clearly distinct from those at day 1 and day 90. Brown adipocyte‐specific genes showed a marked downregulation, whereas white adipocyte markers gradually increased with age. Interestingly, beige adipocyte markers (*TBX1*, *CD137*, *SHOX2*, and *TMEM26*) remained relatively stable between days 14 and 30, suggesting the persistence of a beige‐like transitional phenotype prior to complete whitening.

In this study, we identified two BAT‐specific enhancers of *PGC1A*, designated PGC1A‐En1 and PGC1A‐En2, which exhibit distinct contributions to *PGC1A* regulation and thermogenic activation. In luciferase reporter assays, PGC1A‐En1 displayed markedly stronger enhancer activity than PGC1A‐En2, driving an approximately 4.0‐fold increase in transcriptional activity, whereas PGC1A‐En2 alone produced only a modest 1.4‐fold increase. Consistently, CRISPRi‐mediated repression of PGC1A‐En1 resulted in a substantially greater reduction in *PGC1A* expression compared with repression of PGC1A‐En2, particularly under β‐adrenergic stimulation, where *PGC1A* expression was reduced by 53% upon En1 repression but only 17% upon En2 repression. These results indicate that PGC1A‐En1 functions as the dominant enhancer driving inducible *PGC1A* transcription and thermogenic activation in brown adipocytes, whereas PGC1A‐En2 appears to exert a weaker, context‐dependent modulatory effect. Notably, the simultaneous repression of both enhancers resulted in the most pronounced suppression of *PGC1A* expression and associated thermogenic gene programs. These findings support an additive model of enhancer function [58, 59], wherein PGC1A‐En2 fine‐tunes transcriptional output in concert with the primary enhancer, PGC1A‐En1. Given the central aim of this study to define the key *cis*‐regulatory element with the strongest impact on brown fat thermogenesis, we therefore prioritized PGC1A‐En1 for in‐depth mechanistic dissection and in vivo functional validation. The presence of both a dominant enhancer (PGC1A‐En1) and a secondary enhancer (PGC1A‐En2) suggests a partially redundant regulatory architecture at the *PGC1A* locus. Rather than functioning as independent or mutually exclusive elements, these enhancers appear to cooperate to ensure robust *PGC1A* induction in response to β‐adrenergic signaling, with PGC1A‐En1 providing the primary regulatory drive and PGC1A‐En2 contributing auxiliary input that fine‐tunes transcriptional output. Given the known cellular heterogeneity of brown adipose tissue, it is conceivable that enhancer usage may vary across adipocyte subpopulations or physiological states; however, resolving such context‐specific enhancer activity will require future single‐cell multi‐omics analyses.

Previous studies have highlighted the critical role of conserved noncoding DNA elements in vertebrate development [60]. In this study, we demonstrate that goat PGC1A‐En1 and mouse Pgc1a‐En1 exhibit conservation in sequence, synteny, and epigenomic features. In addition, the goat PGC1A‐En1 sequence identity between human and goat exceeded 80%. PGC1A, a key transcriptional coactivator of UCP1 expression, is rapidly and robustly induced by cold exposure and activates several essential components of the adaptive thermogenic program in brown fat [28]. Although the loss of PGC1A does not affect brown adipocyte differentiation, it markedly reduces the induction of thermogenic genes [30]. Consistent with these observations, we found that inhibition of PGC1A‐En1 or PGC1A‐En1‐En2 significantly decreased the expression of thermogenic genes (*UCP1* and *DIO2*) and mitochondrial genes (*COX5B* and *COX7A1*) in goat brown adipocytes under cold stimulation. Furthermore, PGC1A‐deficient mice exhibited a marked reduction in core body temperature and impaired thermogenic capacity of BAT during acute cold exposure [29, 61, 62]. Similarly, inhibition of the conserved mouse Pgc1a‐En1 under acute cold exposure resulted in a significant reduction in core body temperature and dorsal skin temperature in the interscapular region. Compared with control mice, Pgc1a‐En1 mice displayed increased lipid droplet size and a higher prevalence of monolocular white fat‐like cells in iBAT. Under thermoneutral conditions, BAT thermogenic demand is minimal, and basal mitochondrial function can be maintained by compensatory regulators such as PGC1β [30, 61]. Although repression of Pgc1a‐En1 significantly impairs thermogenic capacity, the short duration of acute cold exposure is likely insufficient to induce detectable changes in mitochondrial morphology. Future studies involving chronic cold stimulation will be required to fully elucidate the contribution of Pgc1a‐En1 to mitochondrial remodeling in brown adipose tissue.

The orphan nuclear receptor ERRA is a regulator of energy homeostasis, mitochondrial biogenesis, and adaptive thermogenesis [47, 63, 64]. CBP, a histone acetyltransferase, facilitates gene expression by interacting with transcription factors and acetylating histones and other proteins [65]. In this study, we demonstrate that the activity of goat PGC1A‐En1 is synergistically enhanced by ERRA and CBP. ERRA overexpression promotes CBP recruitment and increases H3K27ac deposition at the PGC1A‐En1 locus, ultimately elevating *PGC1A* transcription. Our results further revealed that ERRA and CBP synergistically regulate enhancer activity through a highly conserved ERRA‐binding motif2 shared across goat, human, and mouse, in a manner conserved among these species. These findings advance our understanding of the conserved transcriptional regulation of *PGC1A* across species, offering a theoretical foundation and revealing potential molecular targets for developing therapeutic strategies against obesity and related metabolic disorders.

## 4 Conclusion

Our study shows that changes in the transcriptomic and epigenetic landscapes during PRAT whitening downregulate genes essential for maintaining brown adipocyte identity and function, thereby facilitating the transition from BAT to WAT. Moreover, we identify a functionally conserved enhancer essential for *PGC1A* expression and thermogenesis in brown fat. A highly conserved ERRA‐binding motif2 shared by goat, human, and mouse mediates the synergistic regulation of enhancer activity by ERRA and CBP.

## 5 Experimental Section

### 5.1 Sample Collection

Meigu goats were raised at the Meigu Goat Breeding Farm in Liangshan, China (altitude ∼1920 m; 103.03°E, 28.18°N). The animals were fed a standard diet (forage to concentrate ratio, 70:30) twice per day at 07:00–09:00 and 16:00–18:00 and water ad libitum. Female Meigu goats of similar age and physiological condition were selected at four developmental stages: day 1 (birth day), day 14 (14 days after birth), day 30 (30 days after birth), and day 90 (90 days after birth). Perirenal adipose tissue (PRAT) was collected immediately after euthanasia, rapidly snap‐frozen in liquid nitrogen, and stored at −80°C.

### 5.2 Morphological and Histological Study

Goat PRAT or mouse interscapular brown adipose tissue (iBAT) samples were fixed in 4% paraformaldehyde and subsequently embedded in paraffin. Fixed tissues were sectioned into 5 µm slices and subjected to hematoxylin and eosin (H&E) staining.

For immunohistochemistry (IHC) staining of goat PRAT, sections were deparaffinized, rehydrated, and subjected to heat‐mediated antigen retrieval in citrate buffer (pH 6.0). Sections were blocked with 3% bovine serum albumin (BSA) at room temperature (RT) for 30 min, then incubated overnight at 4°C with a rabbit anti‐UCP1 primary antibody (Servicebio, GB112174, 1:100). After washing, sections were incubated for 50 min at RT with an HRP‐conjugated goat anti‐rabbit IgG secondary antibody (Servicebio, GB23303, 1:500). Color was developed using 3,3’‐diaminobenzidine and nuclei were counterstained with hematoxylin.

For immunofluorescence staining of mouse iBAT, sections were blocked with 3% BSA at RT for 30 min. For double labeling, sections were incubated overnight at 4°C with a mixture of primary antibodies: anti‐UCP1 (Servicebio, GB112174, 1:100) and anti‐PGC1A (Proteintech, 66369‐1‐Ig, 1:1000). After washing, sections were incubated with Cy5‐conjugated Goat Anti‐rabbit IgG (Servicebio, GB27303, 1:400) and Cy3‐conjugated Goat Anti‐mouse IgG (Servicebio, GB21301, 1:300) for 50 min at RT. Nuclei were counterstained with DAPI (Beyotime, Shanghai, China) for 10 min at RT.

### 5.3 RNA‐seq Library Construction and Sequencing

RNA‐seq was performed on three biological replicates of goat PRAT collected at postnatal days 1, 14, 30, and 90. Samples with RNA integrity numbers greater than 8 were selected for RNA‐seq. The mRNA was purified from total RNA using poly‐T oligo‐attached magnetic beads. Sequencing libraries were constructed using the VAHTS Universal V6 RNA‐seq Library Kit for MGI (Vazyme, Nanjing, China). Sequencing was performed on the MGI‐SEQ 2000 platform with pair‐end 150 bp (PE150).

### 5.4 RNA‐seq Data Analysis

Sequence reads were aligned to the goat reference genome (ARS1) using HISAT2 (v2.2.1), and gene quantification was performed with the featureCounts function from the Rsubread package (v2.8.1). Gene expression levels were normalized as transcripts per million (TPM). Differentially expressed genes (DEGs) were identified using DESeq2 (v1.36.0), with criteria set at |log_2_ fold change| > 1 and a false discovery rate (FDR) < 0.05. Functional enrichment analysis was performed using Metascape (http://metascape.org). MaSigPro (v3.12) was used to analyze dynamic temporal gene expression patterns. Browning capacity was evaluated using the online bioinformatics tool ProFAT (https://github.com/PerocchiLab/ProFAT).

### 5.5 ChIP‐seq Library Construction and Sequencing

ChIP‐seq was performed on two biological replicates of goat PRAT collected at postnatal days 1 and 90, using antibodies against H3K27ac and H3K4me3. Frozen tissues were ground into a fine powder in liquid nitrogen, crosslinked with 2% formaldehyde for 20 min at RT, and quenched with glycine buffer at a final concentration of 0.2 M. After sonication, a portion of the soluble chromatin was stored at −20°C as input DNA, while the remainder was subjected to immunoprecipitation using antibodies against H3K27ac (Abcam, ab4729) or H3K4me3 (Cell Signaling Technology, 9751). ChIP‐seq libraries were constructed using the NEBNext Ultra II DNA library preparation kit for Illumina (NEB, E7645). Sequencing was performed on the Illumina NovaSeq 6000 platform with PE150.

### 5.6 ChIP‐seq Data Analysis

Adapters and low‐quality sequences were removed using Trimmomatic (v0.38), and the cleaned reads were aligned to the goat (ARS1), mouse (mm10), or human (hg38) reference genomes with Bowtie2 (v2.2.5). PCR duplicates were eliminated using Picard tools (v1.90), and peaks were called using MACS2 (v2.1.2) with the default parameters. Pairwise Pearson correlations and Principal Component Analysis (PCA) were conducted using the plotCorrelation and plotPCA tools, respectively. Heatmaps of ChIP‐seq signals were generated with the computeMatrix and plotHeatmap functions from deepTools.

Promoters were identified as regions enriched for H3K4me3. Enhancers were defined as H3K27ac‐enriched regions that did not overlap with H3K4me3‐enriched regions or the extended regions (2.5 kb upstream and 1 kb downstream) of a gene's TSS. Super‐enhancer (SE) peaks were detected using the Rank Ordering of Super‐Enhancers (ROSE) algorithm, based on H3K27ac ChIP‐seq signals with default parameters.

### 5.7 ATAC‐seq Library Construction and Sequencing

ATAC‐seq was performed on three biological replicates of goat PRAT collected at postnatal days 1 and 90. Frozen tissues were ground in liquid nitrogen, and lysis buffer (10 mM Tris‐HCl pH 7.4, 10 mM NaCl, 3 mM MgCl_2_, and 0.1% NP‐40) was added to the powdered samples. The mixture was incubated on ice for 10 min at 4°C with rotation. Cell suspensions were filtered through a 40 µm strainer and washed with cold resuspension buffer (10 mM Tris‐HCl pH 7.4, 10 mM NaCl, and 3 mM MgCl_2_). Approximately 50,000 nuclei were used for the transposition reaction to perform tagmentation. Tn5‐transposed DNA was purified using AMPure DNA magnetic beads, followed by PCR amplification. Libraries were sequenced on the Illumina NovaSeq 6000 platform with PE150.

### 5.8 ATAC‐seq and DHS‐seq Data Analysis

Quality control of raw sequencing data was conducted using Cutadapt (v1.9.1). The trimmed reads were aligned to the goat (ARS1), mouse (mm10), or human (hg38) reference genomes using Bowtie2 (v2.2.5). Mitochondrial reads were filtered out using Samtools (v1.11), and PCR duplicates were removed with Picard tools (v1.90). Chromatin accessibility peaks were identified using MACS2 (v2.1.2) with the options of “–nomodel –shift ‐100 –extsize 200 ‐B –SPMR –keep‐dup = all –q value = 0.05”. Pearson correlation coefficients between replicates were calculated based on read coverage of genomic regions in BAM files. Principal component analysis (PCA) was performed using the multiBigwigSummary and plotPCA functions from deepTools. Heatmaps of ATAC‐seq signals were generated using the computeMatrix and plotHeatmap functions from deepTools.

### 5.9 Differential ChIP‐seq and ATAC‐seq Peaks

Common peaks between replicates for ChIP‐seq and ATAC‐seq were identified using the intersect function of Bedtools (v1.90) with the parameters ‐f 0.5 ‐wa ‐r. Differential regions of H3K27ac, H3K4me3, and chromatin accessibility between postnatal day 1 (BAT) and day 90 (WAT) were analyzed using edgeR (v4.6.3). Differential peaks were defined using thresholds of |log_2_ fold change| > 0.58 and FDR < 0.05. Enrichment of transcription factor (TF) motifs in differentially accessible ATAC‐seq peaks was conducted using the findMotifsGenome.pl script function of HOMER.

### 5.10 4C‐seq Library Construction and Sequencing

4C‐seq was conducted on goat PRAT collected at postnatal days 1 and 90, and on iBAT from 8‐week‐old male C57BL/6J mice (n = 2 biological replicates). Samples were pulverized in liquid nitrogen, fixed with 2% formaldehyde for 30 min at RT, and quenched with 0.2 M glycine. Crosslinked cells were lysed in lysis buffer for 15 min on ice. Chromatin was digested overnight at 37°C with the primary restriction enzyme *Dpn*II (New England Biolabs), followed by ligation overnight at 16°C using T4 DNA ligase (New England Biolabs). After reversing crosslinks, DNA was further digested overnight at 37°C with the secondary restriction enzyme *Csp6*I (Thermo Fisher Scientific) and ligated again overnight at 16°C with T4 ligase. For library preparation, 4 µg of input DNA was used for 4C PCR, divided into 20 parallel reactions. PCR products were separated on a 2% agarose gel, and DNA fragments ranging from 200 to 800 bp were excised and purified. The 4C‐seq libraries were sequenced on the Illumina NovaSeq 6000 platform with PE150. The primers used for 4C‐seq library construction are provided in Table S9.

### 5.11 4C‐seq Data Analysis

Sequencing reads were demultiplexed based on the viewpoint reading primer sequence. The reading primer sequences were trimmed from sequencing reads, which were then aligned to the goat (ARS1) or mouse (mm10) reference genomes using Bowtie2 (v2.2.5). The generated SAM files were converted to BAM files, sorted, and indexed using Samtools (v1.11).

### 5.12 SVF Cells Isolation, Culture, and Differentiation

Stromal vascular fraction (SVF) cells were isolated from the PRAT of goats at postnatal days 1 and 90. Adipose tissue was minced and digested in 0.1% collagenase type I (Gibco) prepared in HBSS buffer supplemented with 2% pen/strep for 1 h at 37°C. Digestion was terminated by adding an equal volume of DMEM/F‐12 containing 15% FBS and 2% pen/strep. The digested mixture was passed through a 100 µm cell strainer, and the filtrate was centrifuged at 500 g for 5 min to pellet the cells. The SVF cells were then resuspended in complete culture medium (DMEM/F‐12 supplemented with 15% FBS and 1% pen/strep) and seeded into cell culture flasks. For differentiation into brown adipocytes, nearly confluent cell cultures were induced with induction medium containing DMEM/F‐12, 10% FBS, 20 nM insulin, 5 µM dexamethasone, 0.5 mM IBMX, 1 nM T3, and 1 µM rosiglitazone for 2 days. The cells were then maintained in differentiation medium supplemented with DMEM/F‐12, 10% FBS, 20 nM insulin, 1 nM T3, and 1 µM rosiglitazone for another 6 days. For differentiation into white adipocytes, cells were cultured in DMEM/F‐12 with 10% FBS, 20 nM insulin, 5 µM dexamethasone, and 0.5 mM IBMX for 2 days, followed by replacement with DMEM/F‐12 containing 10% FBS and 20 nM insulin every 2 days.

The commercially available immortalized human BAT cell line, hTERT A41hBAT‐SVF (ATCC, CRL‐3385), was cultured in Dulbecco's Modified Eagle's Medium (DMEM) supplemented with 10% FBS, and incubated at 37°C and 5% CO_2_. To induce brown adipocyte differentiation, cells were grown to confluence for 6 days and then treated with an induction medium containing 0.5 µM human insulin, 0.1 µM dexamethasone, 0.5 mM IBMX, 2 nM T3, 30 µM indomethacin, 17 µM pantothenate, and 33 µM biotin for an additional 12 days. The induction medium was replaced every 3 days until cells were harvested.

### 5.13 Identification of Active Enhancers of *PGC1A*

Putative enhancers of *PGC1A* were defined as genomic regions that exhibited obvious chromatin interactions with the *PGC1A* promoter, overlapped with both H3K27ac and ATAC‐seq signals, and were located at least 2 kb from annotated TSSs. Luciferase reporter assay was employed to evaluate the activity of the putative enhancers. The enhancer sequences were PCR amplified from goat genomic DNA and cloned into the Firefly luciferase reporter vector pGL3‐SV40 (Promega). On day 6 of goat brown adipocyte differentiation, cells were cotransfected with the pGL3‐SV40‐enhancer constructs and a Renilla luciferase plasmid (internal control) using Lipofectamine 3000 (Invitrogen). Luciferase activity was measured 48 h post‐transfection using the Dual‐Luciferase Reporter Assay Kit (Vazyme, Nanjing, China). Sequence identity between species was assessed using the NCBI Nucleotide BLAST tool (https://blast.ncbi.nlm.nih.gov/Blast.cgi) with default parameters. The PCR primers used to amplify the putative active enhancers are shown in Table S10.

### 5.14 CRISPR‐Mediated Enhancer Repression

CRISPR sgRNAs were designed using the CRISPOR [66] and CHOPCHOP [67]. Potential off‐target sites were predicted using CRISPR RGEN Tools (http://www.rgenome.net/cas‐offinder/). All the sgRNAs utilized in this study were chosen based on high on‐target activity and low off‐target activity. An additional “g” was appended to the 5′ end of the guide sequence when it did not begin with a “g”, to ensure efficient transcription driven by the U6 promoter.

For transient expression of CRISPR‐dCas9‐KRAB (CRISPRi) system, we utilized the KRAB‐dCas9 expression plasmid (Addgene #112195) [68, 69]. To generate sgRNA expression vectors, synthesized oligonucleotides were annealed and cloned into the BsaI site of the pGL3‐U6‐sgRNA‐PGK‐puromycin vector (Addgene #51133). On day 4 of goat brown adipocyte differentiation, cells were cotransfected with the KRAB‐dCas9 plasmid and sgRNA expression vector using Lipofectamine 3000 (Invitrogen). On day 8, cells were treated with 10 µM isoproterenol (ISO) or vehicle control for 4 h before harvest for experiments.

For stable expression of the CRISPRi system, we utilized the lentiviral vector pLV‐hU6‐sgRNA‐hUbC‐dCas9‐KRAB‐T2a‐GFP vector (Addgene #71237). sgRNA oligonucleotides were synthesized, annealed, and cloned into the BsmBI restriction site. The sequences of all sgRNAs are provided in Table S11.

### 5.15 RNA Isolation and qRT‐PCR

Total RNA was extracted from goat brown adipocytes or mouse iBAT using Trizol reagent (Invitrogen). The RNA was reverse transcribed into cDNA with the HiScript III RT SuperMix (Vazyme, Nanjing, China). Quantitative real‐time PCR (qRT‐PCR) was conducted on a CFX Connect real‐time PCR system (Bio‐Rad, USA) using ChamQ SYBR qPCR Master Mix (Vazyme, Nanjing, China). Relative gene expression was determined using the 2^−ΔΔCT^ method, with *RPLP0* as the internal control. Primer sequences used for qRT‐PCR are provided in Table S12.

### 5.16 Immunocytochemistry and BODIPY 493/503 Staining

Goat brown adipocytes were fixed with 4% paraformaldehyde for 15 min at RT and permeabilized with 0.2% Triton X‐100 for 10 min. After blocking with PBS containing 10% goat serum, cells were incubated overnight at 4°C with primary antibodies, including anti‐PGC1A (Bioss, bs‐42280R, 1:50) and anti‐UCP1 (ABclonal, A5857, 1:100). The next day, cells were incubated with a Cy3‐conjugated Goat anti‐Rabbit IgG secondary antibody (ABclonal, AS007, 1:100) for 1 h at 37°C. Lipid droplets were stained with 5 µM BODIPY 493/503 (Invitrogen) for 20 min at RT, and nuclei were counterstained with DAPI (Beyotime, Shanghai, China) for 5 min at RT. Lipid droplet diameters were quantified using the MRI Lipid Droplets plugin in ImageJ software (v1.53, NIH, USA).

### 5.17 Western Blot

Total protein from goat brown adipocytes or mouse iBAT was extracted using a Total Protein Extraction Kit (BestBio, Shanghai, China). Protein concentrations were quantified using a BCA Protein Quantification Kit (BestBio, Shanghai, China). Equal amounts of protein were separated on a 10% SDS‐polyacrylamide gel and transferred onto PVDF membranes (Millipore). The membranes were blocked with 5% non‐fat milk in TBST for 1 h and subsequently incubated with primary antibodies overnight at 4°C. The primary antibodies used were: anti‐PGC1A (Proteintech, 66369‐1‐Ig, 1:1000), anti‐UCP1 (Cell Signaling Technology, 72298, 1:1000), anti‐Phospho‐p38 MAPK (Thr180/Tyr182) (Cell Signaling Technology, 4511, 1:1000), anti‐p38 MAPK (Cell Signaling Technology, 8690, 1:1000), and anti‐Vinculin (Cell Signaling Technology, 13901, 1:1000). After washing, membranes were incubated with HRP‐conjugated anti‐mouse or anti‐rabbit secondary antibodies for 1 h at 37°C. Protein bands were detected using an ECL detection kit (Beyotime, Shanghai, China) and analyzed with ImageJ software.

### 5.18 Quantification of Mitochondrial DNA Content

Total DNA from brown adipocytes or iBAT was extracted using Animal Tissue DNA Isolation Kit (Foregene, Chengdu, China). Relative mitochondrial DNA (mtDNA) content was determined as ratio of the copy numbers from the mtDNA encoded gene (*Mt‐ND1*) to the nuclear DNA encoded gene (*HK2*) via qPCR. Primer sequences for the qPCR are provided in Supplementary Data 12.

### 5.19 UCSC Genome Browser

The goat PGC1A‐En1 sequence (chr6:43,947,503‐43,948,098; ARS1) was converted to the mouse sequence (chr5:51,591,532‐51,592,232; mm10) using the UCSC Genome Browser liftOver tool [42].

### 5.20 Lentivirus Production and Mice Infection

To produce lentiviral particles, H293T cells were cultured in T175 flasks and transfected at approximately 70% confluence. A total of 95 µg of plasmid DNA, consisting of the transfer plasmid pLV‐hU6‐sgRNA‐hUbC‐dCas9‐KRAB‐T2a‐GFP (Addgene #71237), the packaging plasmid psPAX2 (Addgene #12260), and the envelope plasmid pVSV‐G (Addgene #138479), was mixed at a weight ratio of 4:3:1 and introduced into the cells using a Calcium Phosphate Transfection Kit (Beyotime, Shanghai, China). The culture medium was replaced 8 h post‐transfection, and viral supernatants were collected at 24, 48, and 72 h. The pooled supernatants were concentrated using Microsep Advance Centrifugal Devices with Omega Membrane 100K (PALL). Viral titers were determined using the colloidal gold kit (Biodragon, Beijing, China).

To minimize potential confounding effects arising from developmental adaptation to thermal stress, all mice were bred, raised, and maintained under thermoneutral conditions (30°C) prior to experimentation. Mice were housed in groups under a 12 h light/12 h dark cycle with ad libitum access to food and water. Six‐week‐old male C57BL/6J mice (*n* = 32) were randomly assigned to two groups: a control group receiving dCas9‐KRAB‐GFP lentiviral injections and a treatment group receiving dCas9‐KRAB‐Pgc1a‐En1 lentiviral injections (*n* = 16 per group). Mice were anesthetized via intramuscular injection of 1% sodium pentobarbital. Subsequently, 200 µL of lentiviral suspension (6 × 10^7^ transducing units/mL) was administered bilaterally into the iBAT through four injection sites on each side, following previously established protocols [70]. Lentivirus was administered every three days for a total of four injections, resulting in a cumulative dose of 800 µL per mouse. For acute cold exposure, mice were individually caged and exposed to 4°C for 5 h.

### 5.21 Animal Fluorescence and Infrared Imaging

Animal fluorescence imaging was conducted using the AniView Kirin small animal imaging system (Biolight, Guangzhou, China). Thermal images were obtained by an infrared thermal imaging camera (Fotric 348A, Shanghai, China) and analyzed using Analyz IR software.

### 5.22 Electron Microscopy

Tissue samples were fixed in 2.5% glutaraldehyde (Servicebio, G1102‐100ML) at RT for 2 h. After three washes in 0.1 M phosphate buffer (pH 7.4), the samples were post‐fixed in 1% osmium tetroxide at RT for 2 h. The tissues were then dehydrated through a graded ethanol series, cleared in propylene oxide, and embedded in Epon according to standard protocols. Ultrathin sections (60 nm) were cut, mounted on grids, and sequentially stained with uranyl acetate and lead citrate. Images were acquired using a JEM‐1400 transmission electron microscope (JEOL Ltd., Tokyo, Japan).

### 5.23 Analysis of Transcription Factor Binding Sites

Transcription factor‐binding sites were predicted using the JASPAR database (https://jaspar.genereg.net/) [44], AnimalTFDB v4.0 (http://bioinfo.life.hust.edu.cn/AnimalTFDB4/) [45], and HOCOMOCO (https://hocomoco11.autosome.org/) [46]. The sequence logo for the ERRA‐binding motif was obtained from the JASPAR database.

### 5.24 DNA Pull‐Down Assay

DNA pull‐down assays were conducted using a DNA pull‐down kit (BersinBio, Guangzhou, China) with nuclear lysates from goat PRAT at postnatal day 1. Briefly, the PGC1A‐En1 enhancer region was amplified by PCR using 5′‐desthio biotin‐labeled primers. The desthio biotin‐labeled or non‐labeled DNA was incubated with streptavidin magnetic beads at RT for 30 min with gentle agitation. Then, nuclear proteins were incubated with the DNA‐coupled beads for 1 h at 4°C. The bound complexes were eluted with 60 µL of protein elution buffer for 2 h at 37°C. The silver stain was performed using the Fast Silver Stain Kit (Beyotime, Shanghai, China). Peptides were then analyzed using an UltiMate 3000 RSLCnano System (Thermo Fisher Scientific) coupled to a Q Exactive HF mass spectrometer (Thermo Fisher Scientific).

### 5.25 ChIP Assay

ChIP assays were performed using the ChIP Assay Kit (Beyotime, Shanghai, China). Briefly, goat brown adipocytes at day 5 of differentiation were transfected with the pEGFP‐N1‐ERRA expression vector. On day 8, cells were crosslinked with 2% formaldehyde for 10 min and quenched with 0.125 M glycine for 5 min at RT. The crosslinked chromatin was then sonicated into 200–1000 bp fragments using a Q800R3 Sonicator (QSonica). The specified antibodies were added to the chromatin solution and incubated overnight at 4°C, with approximately 5% of each sample reserved as an input control. Immunoprecipitated complexes were subsequently washed, eluted, and reverse crosslinked. DNA was purified and quantified by qPCR using a CFX Connect Real‐Time PCR Detection System (Bio‐Rad). The antibodies used included anti‐ERRA (Abcam, ab76228), anti‐CBP (Cell Signaling Technology, 7389), and anti‐H3K27ac (Abcam, ab4729). Primer sequences used for qPCR are listed in Table S12.

### 5.26 Construction of Overexpression Vectors and Luciferase Reporter Assay

The coding sequences of ERRA and CBP were PCR amplified from cDNA derived from goat PRAT, mouse iBAT, or H293T cells, respectively. All PCR products were cloned into the pEGFP‐N1 vector via *Kpn*I (New England Biolabs) restriction sites. H293T cells were seeded and cotransfected with luciferase reporter constructs (PGL3‐SV40‐PGC1A‐En1, PGL3‐SV40‐Pgc1a‐En1, or PGL3‐SV40‐PGC1A‐hEn1), Renilla vector, and pEGFP‐N1‐ERRA and/or CBP expression vectors using Lipofectamine 3000 (Invitrogen). At 48 h after transfection, firefly and Renilla luciferase activities were measured using the Dual‐Luciferase Reporter Assay System. Primer sequences used for vector construction are shown in Table S10.

### 5.27 Statistical Analysis

All data are presented as mean ± SEM unless otherwise noted in the figure legends. Statistical analyses were performed using two‐tailed unpaired Student's *t*‐tests or one‐way ANOVA followed by Tukey's post hoc test for multiple comparisons. For all experiments, *p* < 0.05 was considered significant (*p < 0.05, **p < 0.01, *** *p* < 0.001).

## Author Contributions

L.W. and D.S. conceived and supervised the experiments. D.S., H.Y., and X.L. conducted the experiments and analyzed the data. T.J., S.Z., T.Z., and J.G. assisted with animal work. L.W. was responsible for funding acquisition and project administration. Y.W., L.L., and H.Z. helped with data analysis. D.S., H.Y., and X.L. drafted and revised the manuscript. All authors reviewed and approved the final version.

## Ethics Statement

All animal experiments were conducted according to the Regulations for the Administration of Affairs Concerning Experimental Animals (Ministry of Science and Technology, China, revised in March 2017) and approved by the Animal Ethical and Welfare Committee (AEWC) of Sichuan Agricultural University (Approval No. 20240534).

## Conflicts of Interest

The authors declare no conflicts of interest.

## Supporting information




**Supporting File 1**: advs74941‐sup‐0001‐SuppMat.docx.

**Supporting File 2**: advs74941‐sup‐0002‐FigureS1‐S8.zip.

**Supporting File 3**: advs74941‐sup‐0003‐TableS1‐S13.zip.

## Data Availability

The raw sequence data reported in this paper have been deposited in the Genome Sequence Archive (Genomics, Proteomics & Bioinformatics 2021) in National Genomics Data Center (Nucleic Acids Res 2022), China National Center for Bioinformation / Beijing Institute of Genomics, Chinese Academy of Sciences (GSA: CRA028481) that are publicly accessible at https://ngdc.cncb.ac.cn/gsa/. Detailed information about the public datasets used in this study can be found in Table S13.
