# Supplementary material for: A Functionally Conserved Enhancer is Critical for PGC1A Expression and Thermogenesis in Brown Fat
Source: Adv Sci (Weinh). 2026 Mar 23;13(31):e18153. doi: 10.1002/advs.202518153 (PMC13588000; doi:10.1002/advs.202518153)
Supplement: Supplementary file 1 [file ADVS-13-e18153-s003.docx]

Supplementary Information

**A Functionally Conserved Enhancer is Critical for *PGC1A* Expression and Thermogenesis in Brown Fat**

Duo Su^1,2,3^, Haosen Yang^1,2,3^, Xiaokai Li^4,5^, Tingting Jiang^1,2,3^, Siyuan Zhan^1,2^, Tao Zhong^1,2^, Jiazhong Guo^1,2^, Yan Wang^1,2^, Li Li^1,2^, Hongping Zhang^1,2^, Linjie Wang^1,2,3*^

1. Key Laboratory of Livestock and Poultry Multi-omics, Ministry of Agriculture and Rural Affairs, College of Animal Science and Technology, Sichuan Agricultural University, Chengdu, Sichuan, P. R. China.

2. Farm Animal Germplasm Resources and Biotech Breeding Key Laboratory of Sichuan Province, College of Animal Science and Technology, Sichuan Agricultural University, Chengdu, Sichuan, P. R. China.

3. Key Laboratory of Agricultural Bioinformatics, Ministry of Education, Sichuan Agricultural University, Chengdu, Sichuan, P. R. China.

4. Chongqing Academy of Animal Sciences, Chongqing, China.

5. National Center of Technology Innovation for Pigs, Chongqing, China.

Duo Su, Haosen Yang, and Xiaokai Li contributed equally to this work.

* Corresponding author: Linjie Wang

E-mail address: wanglinjie@sicau.edu.cn

Phone: +86-28-86291010

Fax: +86-28-86290987

**Figure S1.** Transcriptional changes associated with PRAT whitening. A) Pearson correlation heatmap showing gene expression profiles of all 12 samples. B) Volcano plot of differentially expressed genes (DEGs, |log_2_ fold change| > 1, FDR < 0.05) at different developmental stages. C) Number of DEGs at different developmental stages. D) Top 20 significantly enriched Gene Ontology Biological Process (GO-BP) terms at different developmental stages.

**Figure S2.** The quality control for ChIP-seq data. A) Scatter plots showing the correlation between biological replicates of H3K4me3 and H3K27ac ChIP-seq in brown adipose tissue (BAT) and white adipose tissue (WAT). B) Heatmaps showing H3K4me3 and H3K27ac ChIP-seq signals within ± 5 kb from the TSSs. C) Distribution of H3K4me3 and H3K27ac ChIP-seq peaks across different genomic regions, including promoter, 5′ UTR, 3′ UTR, exon, intron, downstream, and distal intergenic regions. D) Venn diagram showing overlap between genes downregulated at both the transcriptional and chromatin levels (H3K4me3 and H3K27ac) in BAT compared to WAT.

**Figure S3.** The quality control for ATAC-seq data. A) Fragment length distribution of ATAC-seq reads. B) Scatter plots showing the correlation between biological replicates of ATAC-seq in BAT and WAT. C) Heatmaps showing ATAC-seq signals within ± 5 kb from the TSSs. D) Distribution of ATAC-seq peaks across different genomic regions, including promoter, 5′ UTR, 3′ UTR, exon, intron, downstream, and distal intergenic regions.

**Figure S4.** Construction and quality control of the 4C-seq library. A) Viewpoint selection and primer design at the *PGC1A* promoter region between 2,000 bp upstream to 500 bp downstream of the transcription start site for the 4C-seq experiment. B) Bar plots showing the percentage of mapped reads in *cis*-chromosome and *trans*-chromosome for each 4C dataset.

**Figure S5.** PGC1A-En1 and PGC1A-En2 regulate *PGC1A* expression in goat brown adipocytes. A) qRT-PCR analysis of *PGC1A* expression during the differentiation of goat brown adipocytes (n = 3). B) Amplification (left) and melting (right) curves of *RPLP0* and *dCas9* in goat brown adipocytes after ISO treatment. C) qRT-PCR analysis of *PGC1A*, *UCP1*, and *DIO2* expression in goat brown adipocytes after ISO treatment (n = 6). D-F) qRT-PCR analysis of *PGC1A*, *UCP1*, *DIO2*, *COX5B*, and *COX7A1* expression in goat brown adipocytes (n = 6). G, H) qPCR analysis of mtDNA content in goat brown adipocytes treated with or without ISO (n = 6). Data are presented as mean ± SEM. For statistical analysis, two-tailed unpaired Student’s *t*-tests were performed in C; one-way ANOVA plus Tukey’s post hoc tests were performed in D-H. * *p* < 0.05, *** *p* < 0.001.

**Figure S6.** Repression of Pgc1a-En1 did not significantly affect BAT thermogenic capacity in mice under thermoneutral conditions. A) Viewpoint selection and primer design at the *Pgc1a* promoter region between 2,000 bp upstream to 500 bp downstream of the transcription start site for the 4C-seq experiment. B) Bar plots showing the percentage of mapped reads in *cis*-chromosome and *trans*-chromosome for each 4C dataset. C) Representative images of iBAT in mice two weeks after injection with dCas9-KRAB-GFP lentivirus. GFP (green), PGC1A (red), and DAPI (blue). Scale bar = 50 μm. D, E) qRT-PCR analysis of *Pgc1a*, *Ucp1*, and *Dio2* expression in iBAT under thermoneutral conditions (n = 8). F) Immunofluorescence staining of iBAT for PGC1A (red), UCP1 (yellow), and nuclei (blue) under thermoneutral conditions. Scale bar = 50 μm. G) Western blot analysis of PGC1A and UCP1 in iBAT under thermoneutral conditions (n = 6). Gray values of protein bands were quantified using Image J software, with Vinculin as a normalizer. H) Body weight of mice under thermoneutral conditions (n = 8). I) Core body temperature of mice under thermoneutral conditions (n = 8). J) Representative images of the dorsal skin (left) and quantification of average dorsal skin temperature (right) under thermoneutral conditions (n = 8). K) Representative H&E staining of iBAT under thermoneutral conditions. Scale bar = 50 μm. L, M) qPCR analysis of mtDNA content in iBAT under thermoneutral conditions or after acute cold exposure (n = 8). Data are presented as mean ± SEM. For statistical analysis, two-tailed unpaired Student’s *t*-tests were performed in D-J, L, M. * *p* < 0.05, *** *p* < 0.001.

**Figure S7.** PGC1A-hEn1 regulates *PGC1A* expression in human brown adipocytes. A-C) qRT-PCR analysis of *PGC1A*, *UCP1*, *DIO2*, *COX5B*, and *COX7A1* expression in human brown adipocytes (n = 6). D) qPCR analysis of mtDNA content in human brown adipocytes (n = 6). Data are presented as mean ± SEM. For statistical analysis, two-tailed unpaired Student’s *t*-tests were performed in A. ** *p* < 0.01.

**Figure S8.** Uncropped high resolution scan of blots.

**Table Captions for Table S1-13**

**Table S1.** Sequencing data summary (Upload separately).

**Table S2.** Differentially expressed genes between indicated age groups in goat PRAT (Upload separately).

**Table S3.** Differentially enriched ChIP-seq peaks between BAT and WAT of goats (Upload separately).

**Table S4.** *Cis*-regulatory elements in BAT and WAT of goats (Upload separately).

**Table S5.** Super-enhancers in BAT and WAT of goats (Upload separately).

**Table S6.** Differentially ATAC-seq peaks between BAT and WAT of goats (Upload separately).

**Table S7.** The quality metrics of 4C-seq data (Upload separately).

**Table S8.** Mass spectrometry of goat PGC1A-En1 pulldown proteins (Upload separately).

**Table S9.** The PCR primers for 4C-seq library construction (Upload separately).

**Table S10.** The PCR primers of constructed vectors (Upload separately).

**Table S11.** CRISPRi sgRNA for targeting active enhancers (Upload separately).

**Table S12.** The primers for gene expression, ChIP-qPCR, and mtDNA copy number (Upload separately).

**Table S13.** The detailed information of ChIP-seq, ATAC-seq, and DHS-seq data (Upload separately).
